# Advanced bioinformatic analysis and pathway prediction of NSCLC cells upon cisplatin resistance

**DOI:** 10.1038/s41598-021-85930-y

**Published:** 2021-03-22

**Authors:** A K M Nawshad Hossian, Fatema Tuz Zahra, Sagun Poudel, Camille F. Abshire, Paula Polk, Jone Garai, Jovanny Zabaleta, Constantinos M. Mikelis, George Mattheolabakis

**Affiliations:** 1 School of Basic Pharmaceutical and Toxicological Sciences, College of Pharmacy, University of Louisiana Monroe, Monroe, LA USA; 2 Department of Pharmaceutical Sciences, School of Pharmacy, Texas Tech University Health Sciences Center, Amarillo, TX USA; 3 Louisiana State University Health Sciences Center, Shreveport, LA USA; 4 Stanley S. Scott Cancer Center, Louisiana State University Health Sciences Center, New Orleans, LA USA; 5 Department of Pediatrics and Stanley S. Scott Cancer Center, Louisiana State University Health Sciences Center, New Orleans, LA USA

**Keywords:** Next-generation sequencing, RNA sequencing, Cancer genomics, Cancer models, Lung cancer, Non-small-cell lung cancer, Computational biology and bioinformatics, Cellular signalling networks

## Abstract

This study aims to identify pathway involvement in the development of cisplatin (cis-diamminedichloroplatinum (II); CDDP) resistance in A549 lung cancer (LC) cells by utilizing advanced bioinformatics software. We developed CDDP-resistant A549 (A549/DDP) cells through prolonged incubation with the drug and performed RNA-seq on RNA extracts to determine differential mRNA and miRNA expression between A549/DDP and A549 cells. We analyzed the gene dysregulation with Ingenuity Pathway Analysis (IPA; QIAGEN) software. In contrast to prior research, which relied on the clustering of dysregulated genes to pathways as an indication of pathway activity, we utilized the IPA software for the dynamic evaluation of pathway activity depending on the gene dysregulation levels. We predicted 15 pathways significantly contributing to the chemoresistance, with several of them to have not been previously reported or analyzed in detail. Among them, the PKR signaling, cholesterol biosynthesis, and TEC signaling pathways are included, as well as genes, such as PIK3R3, miR-34c-5p, and MDM2, among others. We also provide a preliminary analysis of SNPs and indels, present exclusively in A549/DDP cells. This study's results provide novel potential mechanisms and molecular targets that can be explored in future studies and assist in improving the understanding of the chemoresistance phenotype.

## Introduction

Lung cancer (LC) represents ~ 13% of all new cancer cases and is the leading cause of cancer-related deaths worldwide^1^. More than 50% of the lung cancer cases are diagnosed in the advanced disease stage (stage III/IV), which leads to a poor survival rate^1,2^. LC subdivides into two main categories, small cell lung cancers (SCLC), which account for ~ 15% of the LC cases, and non-small cell lung cancers (NSCLC), which account for the remaining ~ 85% of the LC cases. The latter can further be divided, with the three more frequently observed groups being adenocarcinomas (~ 40%), squamous cell carcinomas (~ 30%), and large cell undifferentiated carcinomas (~ 15%)^1,3^.

In the last decades, significant progress has taken place on the development of impactful cancer therapeutics for the treatment of NSCLC. Unfortunately, resistance to chemotherapeutic agents is a frequent hurdle in cancer treatment, which leads to disease relapse with a more aggressive outlook^4,5^. Platinum-based compounds, such as cisplatin (cis-diamminedichloroplatinum (II); CDDP) and carboplatin, are at the forefront of the clinically-used chemotherapeutic approaches against NSCLC^5,6^, as well as for head and neck, brain, gastrointestinal, and ovarian cancers^7,8^.

CDDP's primary mechanism is to bind with a cell’s DNA, primarily at the nucleophilic N7 site of purines, inducing DNA damage, cell cycle arrest, and subsequent apoptosis^9–11^. Over prolonged treatment, the development of platinum-based chemoresistance takes place, which has been associated with changes in gene expression and pathway activities^12^, including from non-coding genes, such as miRNAs^13,14^.

Recent developments on bioinformatic analyses allow for better interpretation of gene expression profiles, as identified by RNA-seq analyses. Ingenuity Pathway Analysis (IPA) software (QIAGEN, Hilden, Germany) is an advanced bioinformatics tool, which can analyze the extent of gene dysregulation (i.e., fold-change) and identify its impact on the pathway and cellular activities, based on build-in scientific literature databases (www.ingenuity.com). This translates to the identification of the most impacted pathways and predictions on cellular functions.

In this study, we developed the CDDP-resistance phenotype of the A549 lung adenocarcinoma cells through prolonged exposure of the cells with the drug to evaluate the gene expression alterations and pathway activation. We performed Next Generation miRNA and mRNA sequencing on the parent A549 cell line and the CDDP-resistance phenotype and analyzed the differential expression template.

Unlike previous reports that used gene clustering into pathways to hierarchically sort them by the number of the dysregulated genes, we used advanced bioinformatics based on IPA to evaluate the gene expression dysregulation levels and predict their impact on the activation status of cellular pathways and functions. Our study's main objective is to compare and expand upon the existing literature and share the raw data with the academic community for evaluation, utilization, and potential identification of new targets for future research applications. To this end, we used IPA to evaluate affected, or predicted-as-affected pathways associated with coding or non-coding genes and compared them to the literature.

## Results

### A549 LC cells developed resistance after continuous and prolonged exposure to CDDP

**Figure 1:**
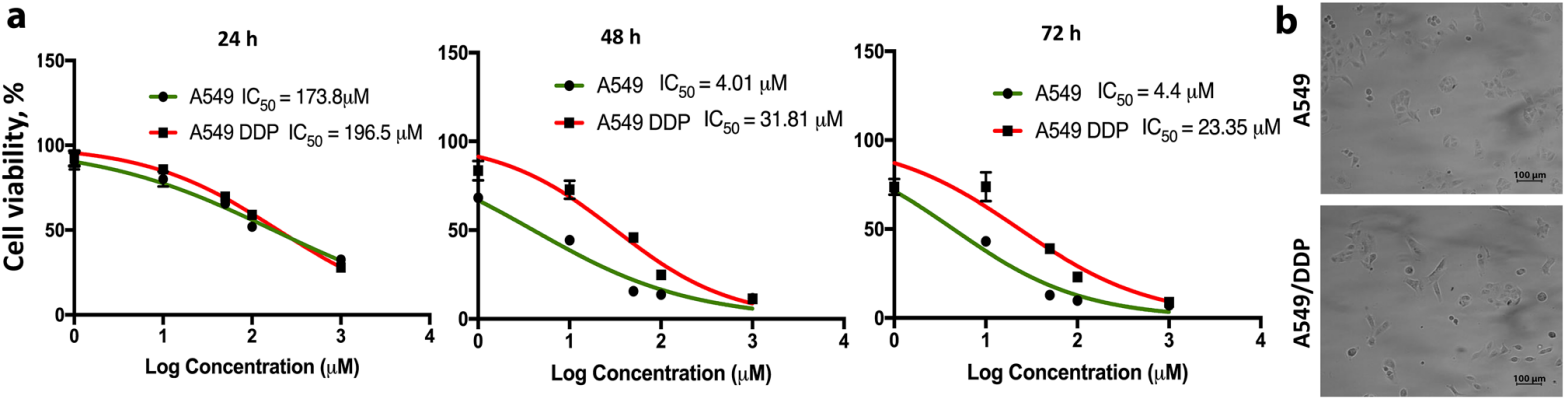
Cytotoxicity analysis of CDDP in A549 and A549/DDP cells. (**a**) We determined the IC50 values of CDDP in the resistant and parent A549 cells following 24, 48, and 72 h of incubation. (**b**) Representative pictures of A549 and A549/DDP cell cultures (Scale bar: 100 μm).

We evaluated A549/DDP cells' resistance development vs. the parental cell line against CDDP using a cell viability assay and through observed changes in the inhibitory concentration (IC_50_). We detected that CDDP’s IC_50_ in the parental A549 cell line is 173.8 μM at 24 h, 4.01 µM at 48 h, and 4.4 µM at 72 h of incubation (Fig. 1). In contrast, CDDP's IC50 value for the CDDP pre-treated cells (A549/DDP) increased to 196.5 μM at 24 h, 31.8 µM at 48 h and 23.35 µM at 72 h of incubation. These data indicate the A549/DDP cell line developed a resistant phenotype against CDDP, with ~ 7.9-fold and ~ 5.3-fold increase of the drug’s IC_50_ values compared to the A549 parent cell line at 48 h and 72 h, respectively. We confirmed this assessment with a blinded, independent investigator at Texas Tech University Health Sciences Center (Supplementary Figure S1a). Subsequently, we evaluated whether the cells maintain the CDDP-resistance phenotype by repeating the cell viability assay after 2 weeks of incubation of the A549/DDP cells in the absence of the drug (Supplementary Figure S1b). The drug’s IC_50_ values indicated that the cells had acquired a stable CDDP-resistance phenotype, with the IC_50_ values of the A549/DDP cells remaining significantly higher than the parental A549 cell line. We authenticated the A549/DDP cell line using ATCC’s cell authentication kit (ATCC 135-XV; ATCC). The analysis confirmed that the cells are 100% match with the A549 (Supplementary Figure S2a). Mycoplasma analysis of the two cell lines was negative (Supplementary Figure S2b).

### CDDP-resistance induced changes in the expression of miRNAs—IPA analysis

**Figure 2:**
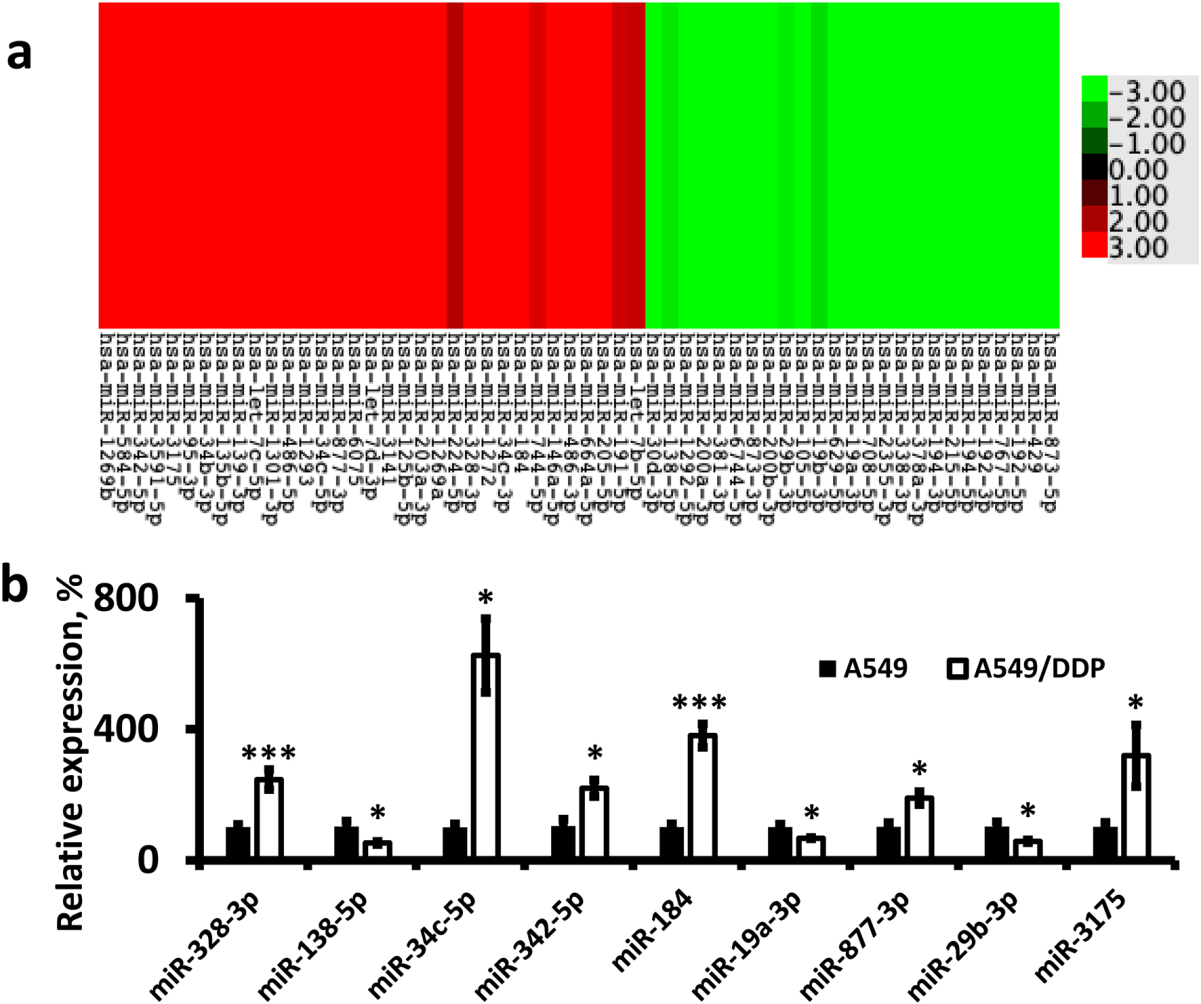
Heatmap of significantly up- and down- regulated miRNAs and representative qPCR analysis of miRNAs. (**a**) Heatmap of differential gene expression, as calculated through RNA-seq of samples extracted from A549 and A549/DDP cells; (**b**) qPCR analysis of representative miRNA expressions. *p < 0.05; ***p < 0.001 for A549/DDP compared to A549.

We performed Illumina miRNA sequencing on RNA samples extracted from A549 and A549/DDP cell lines and determined the differentially expressed miRNAs. We identified 58 miRNAs with altered expression; *p* < 0.05 and false discovery rate (FDR) of < 0.05 (Fig. 2a and Supplementary File 1). In Table 1, we list the top 10 upregulated and downregulated miRNAs. For confirmation, we evaluated the expression of randomly selected miRNAs between A549/DDP and A549, using qPCR (Fig. 2b). The qPCR analysis correlates with the RNA-seq data for all tested miRNAs. Using the IPA software to predict gene targets of miRNAs (Supplementary File 2), we identified 16,197 mRNAs, which are potential targets of 53 miRNAs and are significantly up- or down-regulated in A549/DDP vs. the parent cells. IPA identified that 22 downregulated miRNAs had 3341 upregulated and 2939 downregulated predicted-target genes, while 31 upregulated miRNAs had 4611 downregulated and 5306 upregulated predicted-target genes.

**Table 1:** Top 10 down- and up- regulated miRNAs in A549/DDP vs. A549 cells, as were detected by RNA-seq.

Top downregulated miR		Top upregulated miR	
miR ID	Log_2_FC (A549/DDP vs. A549)	miR ID	Log_2_FC (A549/DDP vs. A549)
hsa-miR-192-5p	− 4.52	hsa-miR-3175	4.97
hsa-miR-767-5p	− 4.47	hsa-miR-146a-5p	4.76
hsa-miR-194-5p	− 4.45	hsa-miR-203a-3p	4.02
hsa-miR-200a-3p	− 4.36	hsa-miR-1269b	3.91
hsa-miR-194-3p	− 3.88	hsa-miR-1269a	3.79
hsa-miR-429	− 3.70	hsa-miR-486-5p	3.43
hsa-miR-192-3p	− 3.50	hsa-miR-486-3p	3.32
hsa-miR-6744-5p	− 3.45	hsa-miR-205-5p	3.13
hsa-miR-873-5p	− 3.43	hsa-miR-3591-5p	3.01
hsa-miR-200b-3p	− 3.37	hsa-miR-328-3p	2.94

### CDDP-resistance changes mRNA expression profile—IPA analysis

**Figure 3:**
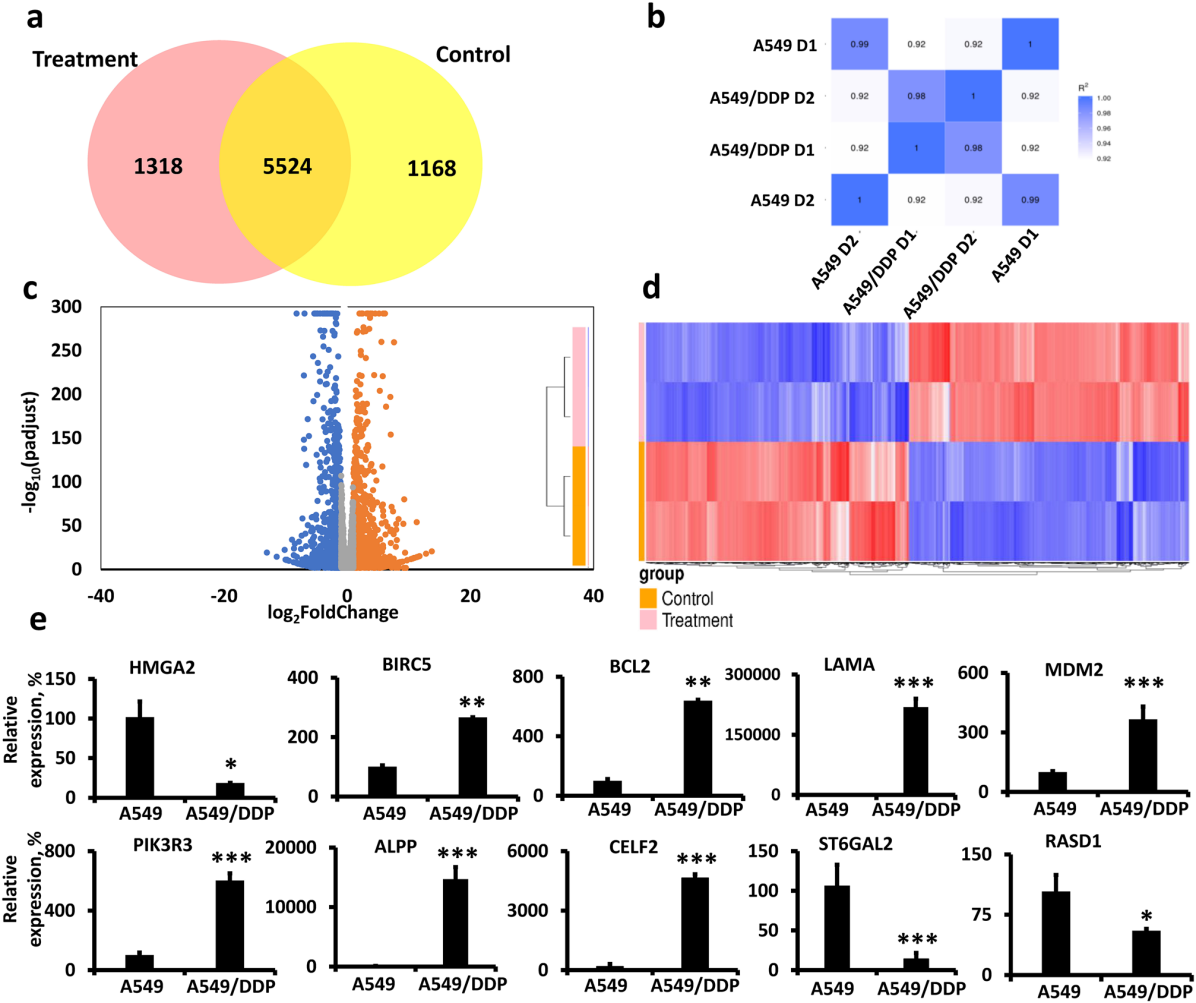
RNA-seq analysis of mRNA extracts from A549 and A549/DDP cells. (**a**) Venn diagram of upregulated genes in control (A549) or treatment (A549/DDP) cells with |Log_2_FC|> 1; (**b**) Pearson Correlation coefficient matrix between samples; (**c**) Volcano graph of gene dysregulations. Colored is for | Log_2_FC|> 1 and FDR < 0.05; (**d**) Heatmap of gene expression; (**e**) qPCR analysis of representative genes. *p < 0.05; **p < 0.01; ***p < 0.001 for A549/DDP compared to A549.

We performed mRNA sequencing to determine differential gene expression between the A549 and A549/DDP. We detected 8010 genes differentially expressed, with FPKM < 1 for all samples being excluded. Among them, 4003 genes were upregulated, and 4007 genes were downregulated (Supplementary File 3; Fig. 3). Briefly, in Fig. 3a, we present a Venn diagram of upregulated genes in the control samples (A549; yellow) or the treatment samples (A549/DDP;  pink) with |log_2_FC|> 1. The overlayed area presents the number of genes that had |log_2_FC|< 1. Figure 3b illustrates the Pearson Correlation coefficient matrix between samples provided by Novogene Co.^15^. Figure 3c presents the volcano graph of gene dysregulations, with orange colored having a log_2_FC > 1 and FDR < 0.05 and blue colored having a log_2_FC < − 1 and FDR < 0.05, and Fig. 3d shows the heatmap of these dysregulated genes. The 10 genes with the strongest up- and down-regulation are presented in Table 2. In Fig. 3e, we present qPCR analyses of HMGA2, BIRC5, BCL2, LAMA, MDM2, PIK3R3, ALPP, CELF2, ST6GAL2 and RASD1, as a confirmation analysis to the RNA-seq data.

**Table 2:** Top 10 down- and up-regulated mRNAs in A549/DDP vs. A549 cells, as were detected by RNA-seq.

Upregulated		Downregulated	
Gene ID	Log_2_FC (A549/DDP vs. A549)	Gene ID	Log_2_FC (A549/DDP vs. A549)
CDH11	13.71	ST6GAL2	− 13.04
LAMA1	12.79	VIL1	− 11.45
CELF2	11.72	USH1C	− 10.16
PCP4	11.46	IGSF11	− 10.03
SPINK6	11.27	A1CF	− 10.01
ADGRF1	11.27	PLPPR1	− 9.862
ALPP	10.90	ANKS4B	− 9.80
S1PR1	10.85	F7	− 9.46
AC091948.1	10.59	PLCXD3	− 8.97
MME	10.33	HAO1	− 8.71

The list of genes in Supplementary File 3 was used for the IPA analysis. As mentioned in the methods section, cutoffs for mRNA expression or pathway activity included: (a) |log_2_(FC)|> 1; (b) − log(*p*) > 1.3, and (c) |z|> 2. For reference, IPA’s z-score indicates a predicted activation or inhibition of a pathway/gene, where a negative z value connotates an overall pathway’s inhibition, and a positive z value connotates an overall pathway’s activation. Values of |z|> 2 are considered significant^16–18^.

**Figure 4:**
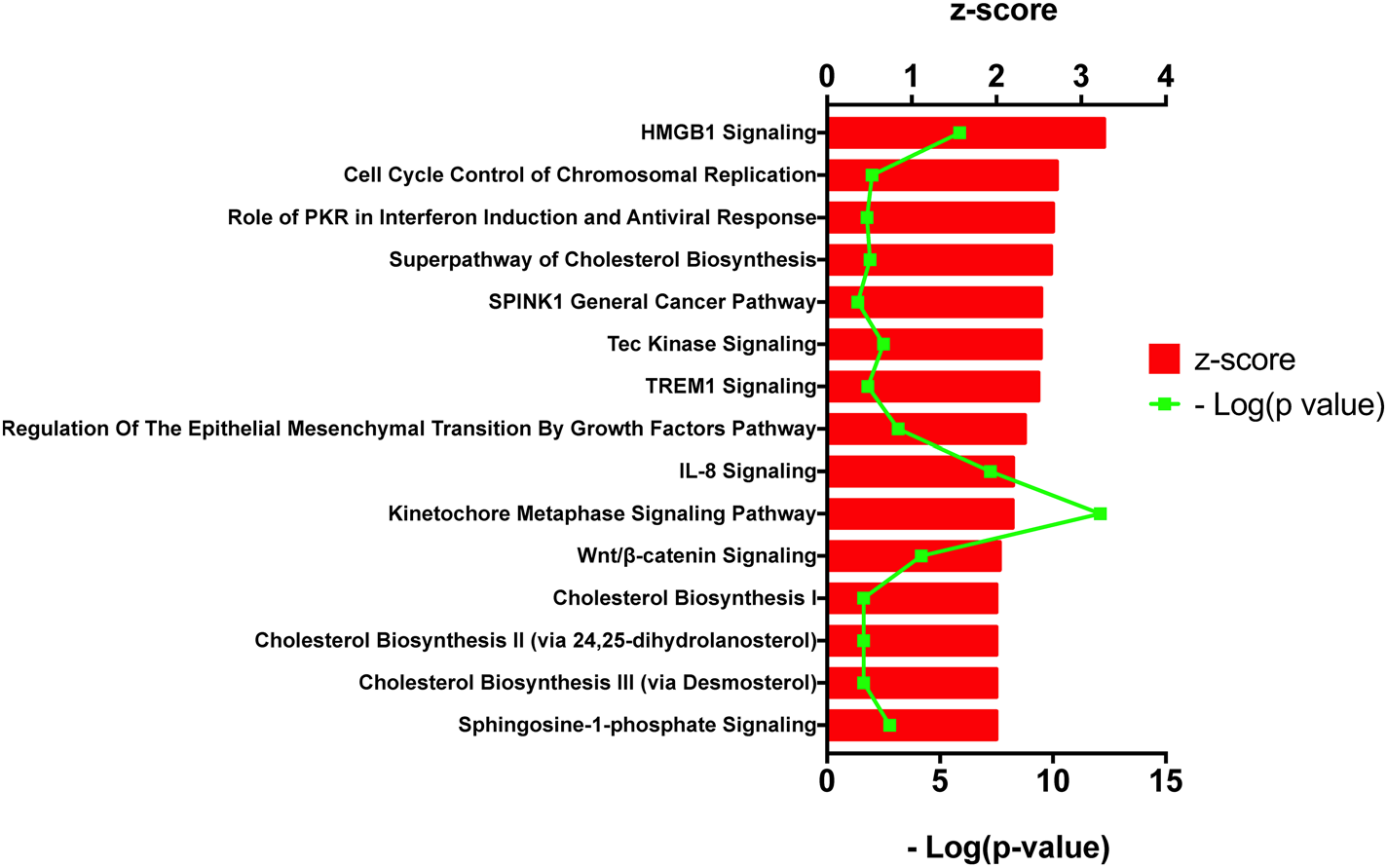
Canonical Pathway analysis. Prediction of activated pathways in A549/DDP cells, with z > 2 and *p* < 0.05 by IPA, based on the differential gene expression compared to A549 cells.

**Figure 5:**
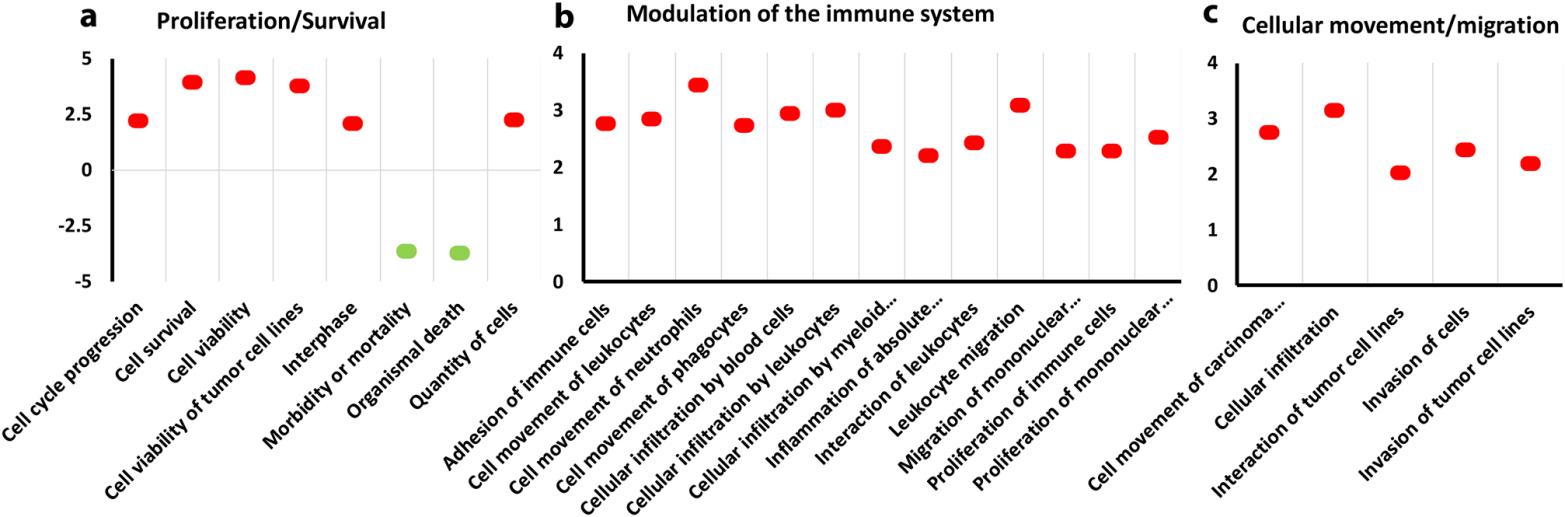
Predicted functions of A549/DDP cells. Bioinformatic analysis predicted functions of the A549/DDP cells based on the RNA-seq data and gene dysregulation level. The functions were categorized in three groups, Proliferation/survival, Modulation of the immune system and Cellular movement/migration. Here shown only functions with |z|> 2 and *p* < 0.05.

The IPA analysis predicted the activation/inhibition of several pathways, out of which, we identified 15 relevant to LC as significantly activated, as shown in Fig. 4 (Supplementary File 4). IPA also predicted 26 cell functions (Fig. 5), based on the detected gene dysregulations and pathway behaviors (Supplementary file 5). For our analysis, we grouped these functions based on their mutual functional characteristics: (1) modulation of the immune system; (2) proliferation/survival, and; (3) cellular movement/migration. Indicatively, in Fig. 5a, for the functions categorized under proliferation/survival, IPA predicted a strong inhibition for the function of “morbidity or mortality” (z = − 3.689; *p* < 0.05). Similarly, in Fig. 5c and under the cell movement category, IPA predicted a strong activation for the function of “Invasion of tumor cells” (z = 2.185; *p* < 0.05). Further analysis of these data takes place in the discussion section.

### Single-Nucleotide-Polymorphism (SNPs) and Indels (insertions–deletions) analysis

**Figure 6:**
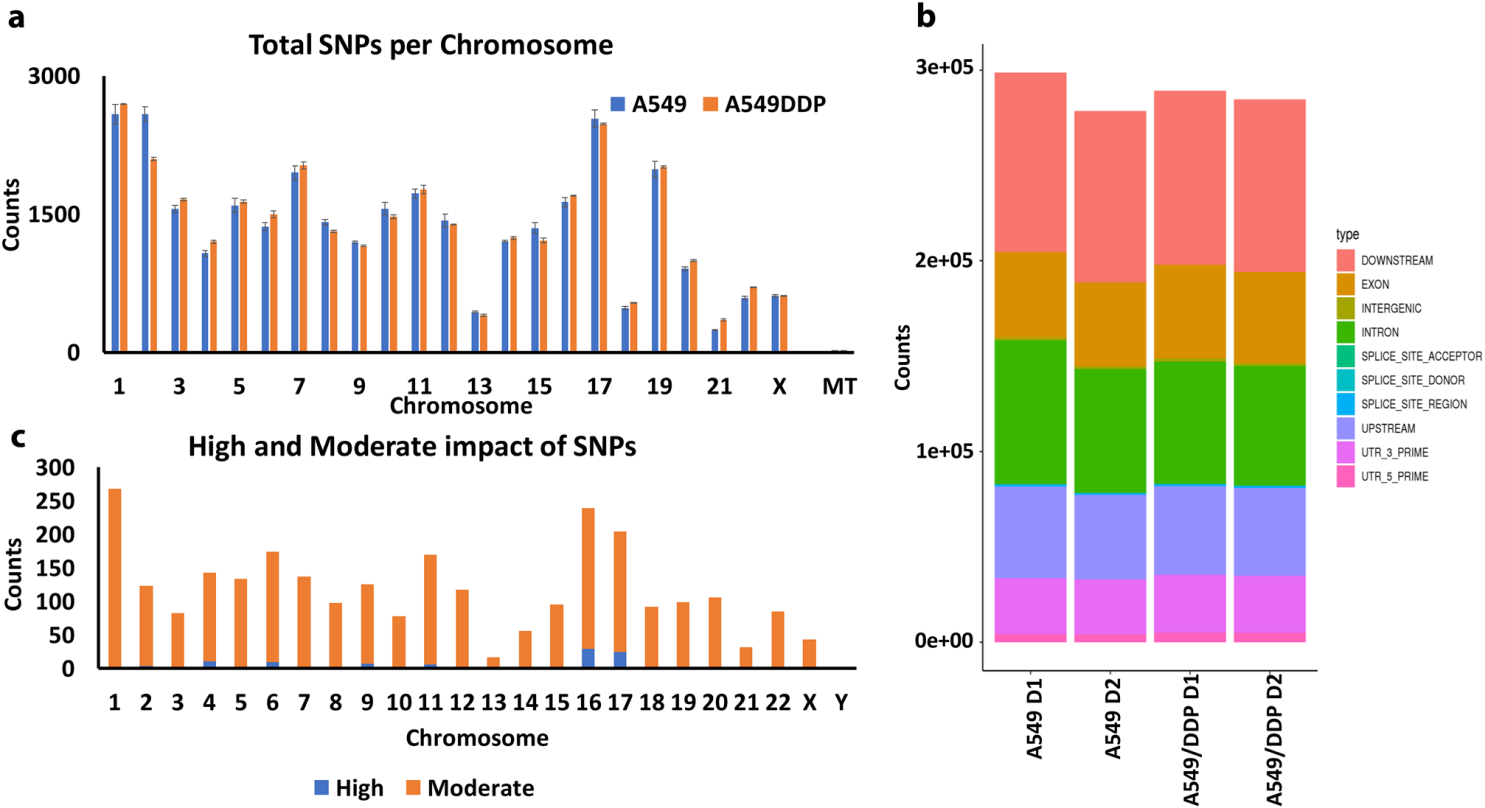
SNP analysis in A549 and A549/DDP cells. (**a**) SNP distribution per chromosome for the two cell lines; (**b**) total number of SNPs per analyzed sample, categorized by type of location, and; (**c**) chromosome distribution of high and moderate SNPs, as classified by snpEff analysis, in A549/DDP cells.

We analyzed the SNPs present in the RNA-seq data from the duplicate samples of A549 and A549/DDP cells to evaluate what SNPs are present in A549/DDP cells following the prolonged treatment with CDDP. These data were further analyzed by snpEff^19^. In Supplementary Table S2, we present the total detected SNPs for each sample and the number of SNP entries located in miRNA genes. In Fig. 6a, we present the average SNPs per chromosome. The SNPs were analyzed using snpEff software to identify SNP entries, genes, and the potential impact of the detected SNPs. In Fig. 6b, we present the type of SNPs (SNP entry), as were categorized by snpEff, for each sample. Briefly, we identified the common SNP entries between the duplicate samples. This yielded 229,072 common SNP entries between the A549 duplicates and 229,134 common SNP entries between the A549/DDP duplicates, based on the chromosomal position. Subsequently, we identified the common SNP entries between the last two groups. This yielded 51,204 SNP entries that were uniquely present in the A549/DDP cells, out of which 211 were located in 129 miRNA genes. Finally, our analysis indicated 102 SNP entries with predicted high impact (according to snpEff analysis) and 2627 SNPs with predicted moderate impact (according to snpEff analysis). In Fig. 6c, we present the SNP entry distribution per chromosome for the SNP entries that were detected only in both of the A549/DDP duplicates.

We analyzed the indel information from the samples. Briefly, we detected 56,812 mutual indel entries (as defined by the snpEff software) between the A549 duplicate samples and 50,648 mutual indel entries between the A549/DDP duplicates. This eventually yielded 15,474 indel entries that were present only in both of the A549/DDP duplicates. Finally, snpEff characterized 401 indel entries with predicted high impact and 43 indel entries with predicted moderate impact. Our analysis does not take into consideration SNPs or indels that were present in A549 and absent in A549/DDP or SNPs that were present in only one of the duplicates of each group.

## Discussion

Chemoresistance is a major challenge in cancer treatment and the cause of failure for various chemotherapeutic approaches^20,21^. The development of chemoresistance involves a complicated interplay of multiple pathways, spanning from alterations in the tumor microenvironment to gene expression alterations in the cancer cells^20,22^. Not surprisingly, multiple genes and pathways participate in the development of chemoresistance. We developed chemoresistance to A549 LC cells using CDDP, a standard of care for LC patients, based on previously reported methodologies^23–25^. Here, we investigated the major gene expression changes that take place following the development of the resistance phenotype, using RNA-seq, and analyzed these changes using the Ingenuity Pathway Analysis (IPA) bioinformatics software.

Unlike previous analyses that relied on gene clustering into pathways to identify affected pathways, IPA predicted, through the level of gene dysregulation, pathway activity (i.e., activation or inhibition) and identified the most statistically significantly impacted pathways. We sought to identify genes and pathways that may not have previously been identified or potentially to present an alternative mechanism for the multifaceted development of chemoresistance in A549 cells.

Initially, we confirmed that the cells developed the CDDP-resistance phenotype (A549/DDP) due to the long-term treatment of the A549 cells with the drug. Using a cytotoxicity assay, we detected the increase of the drug’s IC_50_ value in the A549/DDP cells compared to the parent cell line. For the detection of changes in gene expressions due to the development of the resistance phenotype, we utilized Next Generation Sequencing (NGS) for miRNAs and mRNAs.

Prior to the bioinformatic analysis, we evaluated the differential expression of genes that have been reported in the literature to play a role in the development of chemoresistance of A549 LC cells. For example, in our analysis, ABCB1 (P-gp)^26,27^, WNT5A^27,28^, BIRC5^29^, and BCL2^30^ were upregulated, while HMGA2 was downregulated^31^, which aligned with previous reports. From all the genes detected by RNA-seq, we excluded the genes with FPKM < 1 for all samples. Finally, the genes with FDR < 0.05 and − log(*p*) > 1.3 and |log_2_(FC)|> 1 were used in IPA analysis (total of 2477 genes).

Among the relevant pathways that IPA analysis predicted to be activated, High Mobility Group Protein B1 signaling (HMGB1) had the most potent activation (z = 3.273). HMGB1 is a highly conserved nuclear protein known for its diverse functions, including cell proliferation, differentiation, and death, as well as its involvement in inflammation, angiogenesis, metastasis, immune response, and nuclear stabilization^32–34^. HMGB1 signaling promotes cellular migration and angiogenesis by regulating Receptor for Advanced Glycation End-products (RAGE) and Toll-Like Receptors (TLRs)^35^. Prior literature indicates that upregulation of the HMGB1 protein in A549 cells correlates to the development of resistance phenotype, and downregulation of HMGB1 sensitized the cells to the drug treatment^35–37^. These reports correlate with the IPA’s prediction. In contrast, one report indicates that A549 cells with knocked down HMGB1 were more resistant to drug treatment, though the analysis did not occur with CDDP^38^. Interestingly, the HMGB1 and HMGB2 differential expression according to the raw RNA-seq data (Supplementary File 3) were modestly altered, i.e., HMGB1 was downregulated by 35%, and HMGB2 was upregulated by 35%. In both cases, the |Log2FC| was below 1, and, thus, these gene expressions were not included in the IPA analysis. IPA reached to this prediction based on the overall pathway behavior, which is presented in Supplementary Figure S3.

Our analysis predicted the activation of the cell cycle control and chromosomal replication pathway (Supplementary Figure S4). The activation of this signaling pathway ensures proper cellular developmental events^39^. This pathway includes CDK genes, indicatively CDK1, which has a 2.2-fold increase.

IPA predicted significant activation of Protein Kinase R signaling (PKR; Supplementary Figure S5), which is primarily known for its role in antiviral host defense and the activation of immune responses^40^. The role of PKR signaling in different cancer types, including LC, is not well understood. For example, He et al. demonstrated the high level of PKR caused longer survival of NSCLC patients, whereas Roh et al. presented that higher expression of PKR caused low survival of patients with small-sized peripheral LC^41,42^. In addition, depending on the cancer type and the levels of phosphorylated PKR vs. the expression levels of the protein may also impact the gene’s effect or role on tumor progression^40^. We did not identify relevant research on PKR signaling and A549/DDP cells.

SPINK1 has been associated with poor prognosis in LC patients, and the gene is upregulated in LC tissue samples, compared to the adjacent normal tissues. Similarly, increased expression has been detected in LC cell lines, including A549 cells^43^. SPINK1 also promotes cell growth and metastasis in LC^44^. IPA predicted the activation of the SPINK1 General Cancer Pathway (z = 2.53) in the A549/DDP cells (Supplementary Figure S6). We did not find any prior literature on LC cell and chemoresistance. The RNA-seq data indicated a 10.1-fold increase of SPINK1 expression, though the FDR was 0.063 (i.e., *p* = 0.02), which did not permit the gene to be included in the IPA analyzed data.

TEC Kinase signaling was detected by IPA to be strongly activated (z = 2.524; Supplementary Figure S7). Tec Kinases are a large family of non-receptor tyrosine kinases (nRTK), including Rlk/Txk, Itk/Emt/Tsk, Btk, Tec, and Bmx/Etk^45^. These kinases regulate important pathways, including pathways downstream of G-protein-coupled receptor (GPCR), PTK, TLRs, and integrins, among others^46^. TEC kinases are involved in the progression of several cancer types, including breast, colorectal, and prostate. Additionally, this kinase family is also involved in the regulation of the immune system^45,47^. We did not find prior reports that correlated TEC kinase signaling to cisplatin resistance in LC. Although the gene had a 1.6-fold increased expression, the analysis had FDR of 0.050186, which was slightly out of the FDR limit set above (data not shown).

Epithelial-Mesenchymal Transition (EMT) involvement in the development of drug resistance in LC has previously been described. A549 cells that were resistant following prolonged treatment with gefitinib presented EMT characteristics while demonstrated enhanced invasion and migration potential^48^. It was reported that A549DDP cell resistance correlated to EMT^48^. Interestingly, their analysis relied on the FOXC2 gene, which we did not include in the IPA analysis due to FPKM < 1. Jin et al.^49^ also reported a connection between EMT and the resistance phenotype of A549 cells. The drug-sensitive A549 cells demonstrated a resistant phenotype when treated with TGF-β to induce EMT. Thus, the EMT transition predicted by IPA (Supplementary Figure S8) may constitute a significant target against chemoresistance.

Wnt/β-catenin signaling activation is frequently identified in human malignancies^50^. IPA predicted the activation of Wnt/β-catenin signaling pathway (z = 2.041; Supplementary Figure S9) in A549/DDP cells^27,51,52^. Wnt/β-catenin pathway is an important regulator of many cellular processes, including cell proliferation, development, tumorigenesis, and homeostasis^53,54^. This analysis correlates with several prior studies, which have associated the Wnt/β-catenin pathway activation to chemoresistance through the upregulation of the ABCB1 gene in several cancer types^55,56^, including LC^57,58^. Our RNA-seq data present differentially increased Wnt genes, including WNT5A, WNT7B, and a 120-fold increase of the ABCB1 gene.

IPA also predicted the activation of Sphingosine-1-Phosphate signaling (Supplementary Figure S10). S1P is a natural sphingolipid ligand for the GPCR family, and this signaling molecule carries an emerging role in cellular growth, apoptosis suppression, and immune cell responses^59–61^. It is also a strong regulator of cancer cell proliferation, survival, migration, and angiogenesis^62^. Previous studies suggested that the sphingosine kinase-1 (SPHK1) is a proposed regulator of breast cancer chemoresistance^62,63^, and that cisplatin-resistant NSCLC cells overexpressed SphK1^62^. Interestingly, the RNA-Seq data demonstrated an increase (Log_2_FC = 1.6) of the SPHK1gene, though the information was not included in the IPA analysis due to low FPKM. It also indicated a potent upregulation of the S1PR1 gene expression (Log2Fc = 10.8; *p* = 5.75^–14^; FDR = 5.08^–13^), a GPCR for the SP1 ligand. The expression of S1PR1 has previously been reported to correlate to chemoresistance in neuroblastoma, and its abrogation significantly reduced the observed resistance^64^. Recently, Liu et al.^65^ reported that S1PR1 overexpression promotes proliferation and attenuates apoptosis in esophageal squamous cell carcinomas. To date, we did not find any direct evidence of the S1PR1 and the LC chemoresistance, which may present a promising molecular target for cisplatin resistance in the future. However, it is important to note that careful consideration needs to take place, as there is no evidence of prolonged survival in LC patients with decreased expression of S1PR1.

Cholesterol is increasingly recognized for its critical role in cancer progression. This lipid is a critical component of cell membranes, stabilizing the bilayer while required for cell proliferation. Finally, cholesterol contributes in the organization of lipid rafts, where several signaling cascades take place. Deregulation of cholesterol biosynthesis has been reported in cancer cells and has been connected to cancer progression and resistance^66^. IPA predicted 4 pathways associated with cholesterol biosynthesis that were activated: (a) Superpathway of Cholesterol Biosynthesis; (b) Cholesterol Biosynthesis I; (c) Cholesterol Biosynthesis II (via 24,25-dihydrolanosterol), and; (d) Cholesterol Biosynthesis III (via Desmosterol) (Supplementary Figure S11). Jin et al.^49^ reported on their study on inducing EMT in drug-sensitive A549 cells to develop a resistant phenotype. The effect was reversed by treatment with simvastatin, a drug used to regulate cholesterol metabolism.

The tumor microenvironment has increasingly been recognized for its critical role in tumor onset, progression, metastasis, and resistance^67^. The interaction between tumor cells and the cells of the immune system promote tumor progression, angiogenesis, and metastasis, as well as contributes to the development of chemoresistance^20,68^. The connection between cancer and inflammation has been previously reported, where inflammatory conditions may precede malignancies or tumor cells may promote or inhibit inflammation for tumor progression^69^. The inflammatory responses in the tumor microenvironment (TME) are driven by tumor-associated macrophages, dendritic cells, and other immune system cells that have infiltrated the cancerous tissue^70–72^.

One such case is the Triggering Receptor Expressed on Myeloid Cells-1 (TREM-1) pathway, as IPA predicted its activation (z = 2.496; Supplementary Figure S12). TREM-1 is an important pro-inflammatory innate immune response modulator and is expressed in a subset of myeloid cells, such as tumor-associated macrophages^73^. LC tissue samples have increased TREM-1 expression, though this upregulation occurs in the TME, and TREM-1’s expression correlates to poor prognosis and disease relapse^73,74^. Confirming the existing literature, we did not detect significant TREM-1 expression in A549 cells or A549/DDP cells with RNA-seq^73^. Important regulators of TREM-1 signaling are TNF, GM-CSF, CXCLs, CCL3, CCL20, IL-1β, TLRs, and IL-23, among other^75–78^. We detected an increase in the expression of cytokine genes, such as IL1B (4-fold increase), TLR3 (2.45-fold increase), and TLR4 (56.2-fold increase). This tumor cell behavior during chemoresistance may promote the TREM-1 signaling pathway in cells of the TME through the internal dysregulation of the aforementioned genes. This may also contribute to the previous findings that TREM-1 expression in LC tumors is predictive for cancer aggressiveness and outcome^79^.

Similarly, Interleukin 8 (IL-8; CXCL8) is a chemokine with a potential cancer regulatory effect, as it is used as a biomarker in breast cancer^80^. IL-8 also functions as an autocrine and/or paracrine growth factor for LC development^81^. A previous study suggested that IL-8 promotes chemoresistance in different cancer types, including lung, breast cancer, and ovarian cancer^82–84^. Furthermore, the secretion of IL-8 from tumor cells enhances proliferation and survival through autocrine signaling pathways, as well as activates endothelial cells towards angiogenesis and recruits tumor-associated macrophages to develop a tumor-promoting environment^31^. IPA analysis predicted the significant activation of IL-8 signaling in CDDP-resistant A549/DDP cells (Supplementary Figure S13). RNA-seq detected a 2.6-fold increase in IL8 expression.

Besides pathway analysis, IPA software predicted the A549/DDP cells' functions based on the observed gene dysregulations (Supplementary File 5). We categorized the predicted functions for the A549/DDP cells in three major groups according to similar characteristics/behaviors: a) Immune cell modulation; b) Proliferation/Survival, and; c) Cellular movement or migration. Similar to the pathway analysis, we performed an unrestricted cell-function analysis, which also produced irrelevant results that were rejected. To elaborate, during an unrestricted analysis, the software evaluates changes in gene expression taking place in the tested cells and applies them to any cell type or disease. Though this may present the activation of functions that would not apply in our case (i.e., activation of Pelvic cancer), this approach allows evaluating what would occur in other cells, such as cells of the TME. Understanding that these results present potential behaviors, the existence of the paracrine effect, cell-to-cell communications, and surface receptors’ dysregulations support the implementation of an unrestricted analysis.

First, IPA predicted increased cellular movement for the cancer cells. Chemoresistant cells have been reported to have increased migratory potential in LC^12^ and other types of cancer^85–87^. More specifically, the functions of cellular movement and invasion of tumor cells were predicted to increase. Previous findings confirm that the A549/DDP cells present stronger migration and invasion behavior than the A549 parent cell line^88^.

As we described in the canonical pathway sections, changes in the tumor microenvironment and the behavior of tumor-associated immune cells, such as macrophages, have been associated with the onset and maintenance of chemoresistance^20^. IPA predicted changes in immune responses and functions from the observed A549/DDP cells’ gene dysregulation. The predicted functions should be regarded as an indication for immune responses, which may not necessarily translate to immune cell behavior. IPA predicted a significant activation of functions associated with immune responses, which correlates to significant changes in gene expression/pathway activations, as described above. Briefly, expression changes include the S1PR1 gene, interleukins, CXLs, the Platelet Activating Factor Receptor (PTAFR) gene^89^, and other proteins associated with the interaction of the tumor cells and cells of the immune system. For example, RNA-seq indicated a 72-fold increase of the PTAFR gene in the A549/DDP cells. It has been reported that activated PTAFR-dependent pathways in tumor cells affect the tumor microenvironment and the phenotype of the tumor-associated macrophages to promote tumor growth^90,91^. Of note, we did not find any previous study on PTAFR’s regulatory effect on A549/DDP cells.

Within the category of Proliferation/Survival, IPA predicted a decrease in morbidity and decrease of cellular mortality while predicted the strongest increase for cell viability and survival with z score of approximately 4. This is not surprising, recognizing that the development of chemoresistance is directly linked to the survival of the tumor cells. This is achieved either through the modification of the TME^20^ or gene dysregulations that prevent apoptosis and promote proliferation^92,93^.

We analyzed the sequencing data on miRNA expression in correlation to the data on mRNA expression, using the IPA function to identify miRNA-mRNA targets (Supplementary file 2). Due to the large number of the predicted miRNA-mRNA targets (> 16,000), we narrowed our analysis by selecting only the miRNA-mRNA targets with predicted pathway activity by the IPA, and demonstrated opposite dysregulation (i.e., upregulated miRNA with downregulated mRNA and vice-versa). This included > 3000 miRNA-mRNA targets, which frequently were categorized in more than a single pathway. For example, the analysis indicated that the gene PPM1D (log2(FC):1.724) is a potential target of miR-29b-3p (log2(FC): − 1.494), miR-381-3p (log2(FC): − 1.747), miR-767 (log2(FC): − 4.447) and miR-873-3p (log2(FC): − 3.198), which all were predicted to associate with the AMPK Signaling, ATM Signaling, Cell Cycle: G2/M DNA Damage Checkpoint Regulation pathways. Unfortunately, the relevant keyword of “resistance” in the described pathways only narrowed down the selection to miRNA-mRNA that had the “Cancer Drug Resistance By Drug Efflux” pathway. Nonetheless, it allowed us to perform narrowed down observations. We selected miRNA-mRNA targets (5 of each), where the miRNA had the strongest up-/downregulation, or the mRNA had the strongest up-/downregulation, based on the “Cancer Drug Resistance By Drug Efflux” pathway. In Supplementary File 6—“Top 5 up and down miR” and “top 5 up and down mRNA” tabs, we present these findings. Due to the extensive list of miRNA-mRNAs, below, we describe two representative examples.

We detected a decrease in miR-29b-3p expression. This agrees with a recent report, where the investigators also described a downregulation of miR-29b-3p in A549/DDP, while its upregulation sensitized the cells to cisplatin treatment^94^. Also, increased levels of miR-29b-3p can sensitize osteosarcoma and colorectal cancer cells to methotrexate and oxaliplatin, respectively^95,96^. Moreover, the downregulation of miR-29b-3p induced a chemoresistance phenotype in ovarian cancer cells against cisplatin^97^. IPA predicted PIK3R3 as a significantly upregulated target of miR-29b-3p and also associated with the drug resistance by drug efflux (Supplementary File 6—“Top 5 up and down mRNA” tab). RNA-seq detected a sevenfold upregulation of PIK3R3 in A549/DDP cells. We did not find prior literature on this miRNA’s activity through PIK3R3 in LC cells' cisplatin resistance. However, a previous study suggested the miR-29b reduced cisplatin resistance in gastric cancer by targeting PI3K/AKT pathway^98^.

Similarly, mouse double minute 2 homolog (MDM2) was also identified as a significantly upregulated target of miR-29b-3p downregulation. MDM2 is an important negative regulator of the TP53 tumor suppressor. In a study using seven sensitive/resistant cell line pairs and gene microarrays, MDM2, among other genes, was reported to be associated with the resistance phenotype^99^. We did not find any work on MDM2 and cisplatin resistance in LC.

Next, we found a significant increase of miR-34c-5p (4.95-fold) in A549/DDP cells compared to A549 cells, which IPA predicted related to drug resistance and targeted the RRAS gene. A previous study suggested that reduced expression of miR-34c-5p increased the expression of RRAS^100^. We could not find any direct literature about the role of this miRNA on cisplatin-resistant LC. However, there are conflicting reports regarding this miRNA’s activity in chemoresistance, depending on the cancer type. Catuogno et al.^101^, showed that increased levels of miR-34c-5p markedly contributed to resistance to apoptosis induced by paclitaxel in A549 cells. On the other hand, Tung et al.^102^, showed that the reduced expression of miR-34c-5p leads to increased amphiregulin (AREG) levels responsible for the docetaxel and carboplatin resistance in ovarian cancer. In our data, we observed the decreased expression of the AREG gene.

To better understand the chemoresistance mechanisms, we performed the mutation (SNP and Indel) analysis form RNA-Seq data. A single nucleotide polymorphism (SNP) is a DNA variation, in which a single nucleotide differs between members of a biological species or cells^103^. SNPs can affect gene activity and, hence, the cancer progression and tumor cell behavior to cancer treatment. For example, SNPs may change protein structure and activity that traditional analysis, such as the value of protein expression, may not take into consideration^104^. SNP analysis presents significant challenges, as any gene alterations may take place in areas that are not impactful on a gene’s activity or in exon regions and UTR regions. Furthermore, the larger numbers of SNPs, and gene variants, require extensive and case-by-case analysis to identify any potential effects. Similarly, indels, i.e., the insertion or deletion of bases in the genome, may contribute on the activity of genes^105^. Here, we performed a preliminary analysis of the SNPs and indels between the A549/DDP and A549 cells. The only criteria outcome used here is the presence of chemoresistance (A549/DDP) vs. lack of chemoresistance (A549). Thus, the lack of patient responses, which may indicate the significance of a certain SNPs in a group, and the averaged analysis due to the pooling of mRNA from multiple cells during RNA-extraction, can lead to variations and uncertainty. Following analysis of the SNPs present in the different samples, we identified the SNPs that were present in both A549/DDP duplicate samples but not present in the A549 parental cell line. Of important note, this analysis does not take into consideration any SNPs that were present in A549 cells but absent in A549/DDP cells, neither the SNPs that may have been present in only one of the duplicate samples. For example, the A549 cells’ SNPs for TP53 were at Chr17, positions: (a) 7,674,797 (duplicate 1 and 2); (b) 7,676,154 (duplicate 1 and 2); (c) 7,676,483 (duplicate 1); (d) 7,665,880 (duplicate 2), while for A549/DDP cells’ SNPs for TP53 were at Chr17, position 7,676,154 (duplicate 1 and 2). Such absence of SNPs is not taken into consideration at the moment. SnpEff was used to identify potential impacts of the detected SNPs and indels, which provided with an increased number of SNP and indel entries. Out of the A549/DDP-only SNPs, we selected those that were in genes with |log2(FC)|> 1. These yielded 11,189 SNP entries (Supplementary File 7). Performing the same analysis, we identified 2485 indels only in A549/DDP cells. SnpEff analysis allowed to identify the impact of the SNPs and indels, classified as low, moderate, high or modifier. The relevance of these genes and the respective SNPs/indels to LC resistance merits further evaluation in future studies, with functional validation studies and CRISPR-Cas editing^106^.

Chemoresistance is a complex mechanistic artifact that may manifest due to different pathway activities within the tumor cells or the cells of the tumor microenvironment. This can translate to different pathways having a different impact on the chemoresistance phenotype, which may not necessarily be identical from cell to cell or cancer type. From the analysis above, it was apparent that several pathways, which we report here as potentially activated during chemoresistance, have previously been identified and centrally evaluated individually as key targets for abrogating the phenotype. This adds to the complexity of whether a single pathway manipulation is sufficient or not to abolish the phenotype. Furthermore, we identified additional pathways (i.e., PKR signaling, cholesterol biosynthesis, Tec signaling, among others) or genes (i.e., PIK3r3, miR-34c-5p, MDM2, among other) that may participate in the chemoresistance and merit evaluation in future studies.

## Materials and methods

### Materials

A549 human lung adenocarcinoma cell line was purchased from ATCC (Old Town Manassas, VA). Cisplatin was purchased from Acros Organics (NJ). Cell culture medium DMEM was purchased from VWR (Sanborn, NY), and F12K media was purchased from Corning (Manassas, VA).

### Cell culture

NSCLC A549 cells and the CDDP-resistant A549 cells were cultured in DMEM/F12K complete medium supplemented with 10% fetal bovine serum and 1% penicillin/streptomycin. Cultured cells were maintained at 37 °C with a continuous supply of 5% CO_2_ and humidity^16,17,107^.

### Generation of CDDP-resistant A549 cell line

We used previously published protocols for the development of the CDDP resistance phenotype in A549 cells, with minor modifications^23–25^. Briefly, CDDP-resistant A549 cells (A549/DDP) were generated by continuous incubation in complete media containing CDDP. Initially, A549 cells were incubated with 5 µM of cisplatin, where the cells were maintained on a cycle of 3 days with the drug, followed by 3 days without CDDP (3 days on/off CDDP treatment). After 3 months, the cells were incubated in the presence of 20 µM of CDDP for additionally 3 months, following the same 3 days on/off CDDP cycle.

### Cell survival analysis and authentication

Cell survival analysis was performed using the CellTiter-Fluor Cell Viability Assay (Promega, Madison, WI) fluorescence-based method, following vendor’s instructions. Briefly, we seeded 1 × 10^4^ cells of A549 and A549/DDP in the wells of a 96-well, black, optical bottom plate with complete media. Following overnight incubation at 37 °C, we treated the cells in predetermined wells with CDDP, using serial dilutions of the compound in complete media and incubated for 24, 48, and 72 h. Following confirmation of the resistance phenotype in the A549/DDP cells, we authenticated the cell line using the ATCC human cell line authentication service (Product no: 135-XV). We also performed mycoplasma analysis, following the vendors protocol (Product no: 30-1012 K).

### RNA extraction and qPCR

We performed RNA extraction and qPCR analysis, following previously published protocols^16,17^. Briefly, we extracted total RNA using the Quick-RNA miniprep kit (Zymo Research-Irvine, CA) following the manufacturer’s instruction. We quantified the RNA concentration using Nanodrop, and used the Verso cDNA synthesis kit (Thermo Fisher, Waltham, MA) for cDNA synthesis, according to the manufacturer’s protocol. miRNA cDNA was prepared using TaqMan reverse transcription kit (Applied Biosystem, Carlsbad, CA). We performed quantitative real-time qPCR (RT-qPCR) to detect mRNA and miRNA expression, following the manufacturer’s protocol, using the Bio-Rad CFX96 real-time PCR system (Bio-Rad systems, USA). We used PowerUp SYBR Green Master Mix for mRNA expression and TaqMan Universal Master Mix II assay and TaqMan miRNA specific primers for miRNA expression (Applied Biosystem, Carlsbad, CA). Relative miRNA expression was calculated using the ∆∆Cq analysis. Primers are presented in Supplementary Table S1.

### Next-generation miRNA sequencing

5 µg of RNA were used for the library preparation using the QIA miRNA library kit (QIAGEN, Hilden, Germany). We performed data analysis in the QIAGEN’s GeneGlobe Data Analysis Center software using the embedded DESeq2 normalization algorithm. Raw data (UMIs) were extracted and uploaded to Partek Flow for additional analysis. Differential expression was done with Gene Specific Analysis (GSA) using A549 cells as reference/control, and A549/DDP as treatment.

The normalization method accounts for the miRNA composition population in each sample. It uses a scaling factor to place the molecular tag counts across all samples into the same scale. Each sample’s scaling factor is calculated as the median of the ratios of observed counts to the geometric mean of each corresponding miRNA across all samples. miRNA sequencing was done at the Translational Genomics Core of the Stanley S. Scott Cancer Center, LSUHSC-New Orleans.

### Next-generation mRNA sequencing

Total RNA from A549 and A549/DDP cells was isolated, assessed and processed for sequencing, as previously described with some modification^16,108^. Samples were analyzed by Novogene Co., Ltd. The RNA was evaluated for degradation and contamination and the RNA concentration was assessed. Total of 1 µg of RNA from each sample was used for library preparation. Raw data in FASTQ format were first cleaned using fastp. Reference genome hg38 from NCBI website was used, and paired-end cleaned read of samples were mapped. Read count was performed using FeatureCounts, and differential expression analysis was performed using R with DESeq2 package. *P* values < 0.05 were considered significant. For mutation analysis, Picard tools and Samtools were used to sort, mark duplicate reads, and reorder the bam alignment for each sample. HaplotypeCaller tool was used in GATK software to perform variant discovery. ANNOVAR was used to functionally annotate variants.

### Bioinformatic analysis

We analyzed the pathway behavior due to the differentially expressed genes using the Ingenuity Pathway Analysis software (IPA, QIAGEN), as previously described with some modifications^16^. For the analysis, we removed the gene expressions that have: (a) log2-fold-change (log_2_FC) values between − 1 and 1; (b) *p* > 0.05; (c) False Discovery Rate (FDR) > 0.05, and; (d) FPKM < 1 for all samples. We performed an unrestricted analysis with IPA, meaning we did not define species, cell type, or other characteristics, which may exclude valid results. The authors evaluated any non-relevant results and eliminated them as marked in each respective supplementary data file. The non-relevancy was based on whether the produced result has been reported in the literature to connect to lung cancer or lung cancer chemoresistance. Indicatively, other cancer types (i.e., Pelvic cancer) were excluded from the analysis. The non-relevant results are still presented and marked as such in the supplementary files. The final number of genes analyzed by the IPA software was 2477 mRNAs and 58 miRNAs. All IPA scores of |z|> 2 were considered significant.

## Conclusions

Chemoresistance is a significant hurdle in cancer treatment. Multiple pathways and cellular activities contribute to the manifestation of the phenotype. Here, we developed the CDDP-resistant A549/DDP cells, and used advanced bioinformatics to identify potential pathways that contribute to the resistance. Approximately 15 pathways were described as potentially participating in the development of the chemoresistance, among which several new pathways are presented here for consideration for future in vitro and in vivo studies.

## Supplementary Information

Supplementary Data 1.

Supplementary Data 2.

Supplementary Data 3.

Supplementary Data 4.

Supplementary Data 5.

Supplementary Data 6.

Supplementary Data 7.

Supplementary Information.

## Data Availability

All data generated or analyzed during this study are included in this published article (and its Supplementary Information files).
