# Supplementary material for: Advanced bioinformatic analysis and pathway prediction of NSCLC cells upon cisplatin resistance
Source: Sci Rep. 2021 Mar 22;11:6520. doi: 10.1038/s41598-021-85930-y (PMC7985311; doi:10.1038/s41598-021-85930-y)

# Advanced Bioinformatic Analysis and Pathway Prediction Based on microRNA and mRNA Expression of Cisplatin-Resistant Non-Small Cell Lung Cancer Cells

**A K M Nawshad Hossian[1], Fatema Tuz Zahra[2], Sagun Poudel[1], Camille F. Abshire[3], Paula Polk[3], Jone Garai[4], Jovanny Zabaleta[5], Constantinos M. Mikelis[2], and George Mattheolabakis[1*]**

[1]School of Basic Pharmaceutical and Toxicological Sciences, College of Pharmacy, University of Louisiana Monroe, Monroe, LA, United States
[2]Department of Pharmaceutical Sciences, School of Pharmacy, Texas Tech University Health Sciences Center, Amarillo, TX, United States
[3]Louisiana State University Health Sciences Center, Shreveport, LA, United States
[4]Stanley S. Scott Cancer Center, Louisiana State University Health Sciences Center, New Orleans, LA, United States
[5]Department of Pediatrics and Stanley S. Scott Cancer Center, Louisiana State University Health Sciences Center, New Orleans, LA, United States

## Supplementary Materials

## Supplementary Table S1: Used primers for qPCR analysis

| Gene name | FW primer | RV primer | Amplicon size (bp) |
|---|---|---|---|
| BCL-2 | CCTTGTGGATGACTGAGTACC | AGCCAGGAGAAATCAAACAGAG | 124 |
| HMGA2 | CACTTCAGCCCAGGGACAA | CTCACCGGTTGGTTCTTGCT | 91 |
| BIRC5 | AGAACTGGCCCTTCTTGGAGG | CTTTTTATGTTCCTCTATGGGGTC | 170 |
| CELF2 | CCAGGGTAGGGCTGATAAGG | TGAGTGATCCAAAGCTCCGT | 245 |
| PIK3R3 | CTTGCTCTGTGGTGGCCGAT | GACGTTGAGGGAGTCGTTGT | 164 |
| LAMA1 | AAGTGGCACACGGTCAAGAC | GACAAGAGCTGCATATCCGC | 327 |
| ALPP | CTGCTCTCGGACTCCCTACC | CCTCAAGTCCCACAGGTGAT | 212 |
| ST6GAL2 | TTCCAAAATGCTGAACCCGC | AGAGTTGAGGATTGCGCCTG | 286 |
| MDM2 | GAACTTGGTAGTAGTCAATCAGC | GCCTGATACACAGTAACTTGATA | 534 |
| RASD1 | CCATCGAGGACTTCCACCGC | AGTCGCGGTCACCCTTGTTG | 211 |

**Supplementary Table S2: SNP and indel analysis present in A549 and A549/DDP cells, as were detected by the RNA-seq**

| Sample Name | Total detected SNPs | SNP entries in miRNA genes/# of miRNAs | Total detected indels | Indel entries in miRNA genes/# of miRNAs |
|---|---|---|---|---|
| A549 Replicate 1 | 33,252 | 1,052/335 | 11,061 | 281/157 |
| A549 Replicate 2 | 31,104 | 958/316 | 10,316 | 247/135 |
| A549/DDP Replicate 1 | 32,805 | 1100/358 | 9,663 | 249/144 |
| A549/DDP Replicate 2 | 32,162 | 1068/353 | 9,559 | 242/138 |

**Supplementary Figure S1**. (A) CDDP cytotoxicity analysis in A549 and A549/DDP cells, as performed by blinded investigators; (B) CDDP cytotoxicity analysis in A549 and A549/DDP cells, following lack of A549/DDP cells incubation with CDDP for two weeks.

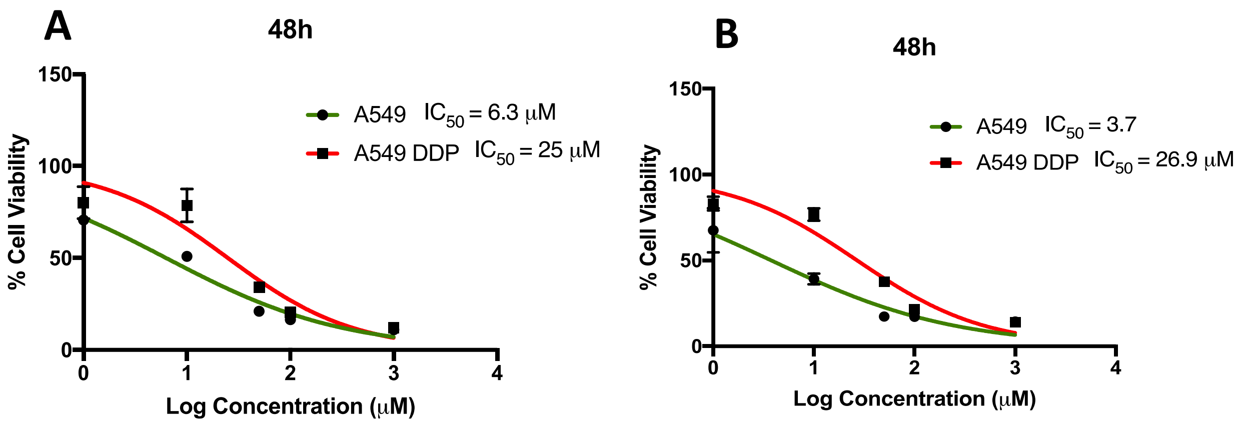

**Supplementary Figure S2**. (A) Cell authentication analysis, as provided by ATCC®; (B) Mycoplasma evaluation of the A549 and A549/DDP cells; (C) Full image of gel picture

A

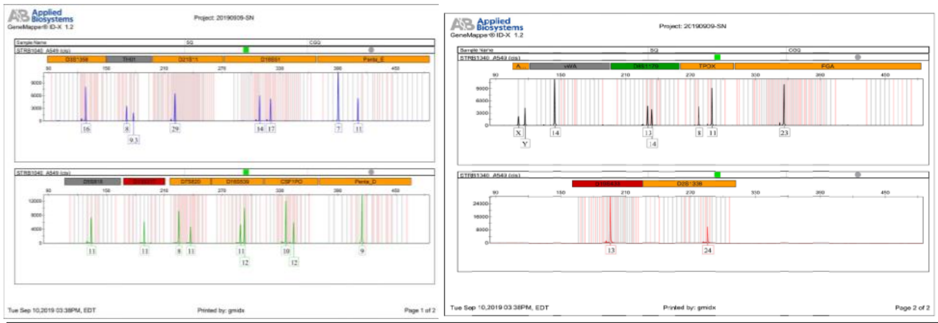

| % Match | ATCC® Cat. No. | Designation | D5S818 | D13S317 | D7S820 | D16S539 | vWA | TH01 | AMEL | TPOX | CSF1PO |
|---|---|---|---|---|---|---|---|---|---|---|---|
| 100 | STRB1040 | A549 (cis) | 11 | 11 | 8,11 | 11,12 | 14 | 8,9.3 | X,Y | 8,11 | 10,12 |
| 100 | CCL-185 | A-549; Lung Carcinoma; Human (Homo sapiens) | 11 | 11 | 8,11 | 11,12 | 14 | 8,9.3 | X,Y | 8,11 | 10,12 |

B

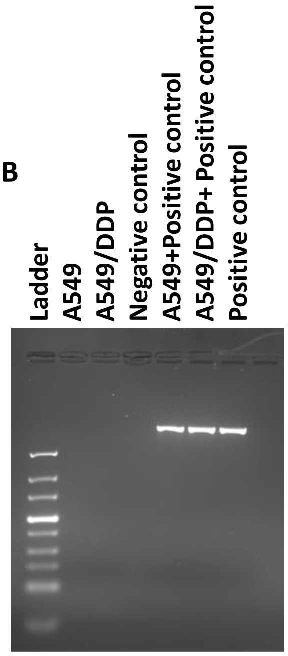


C

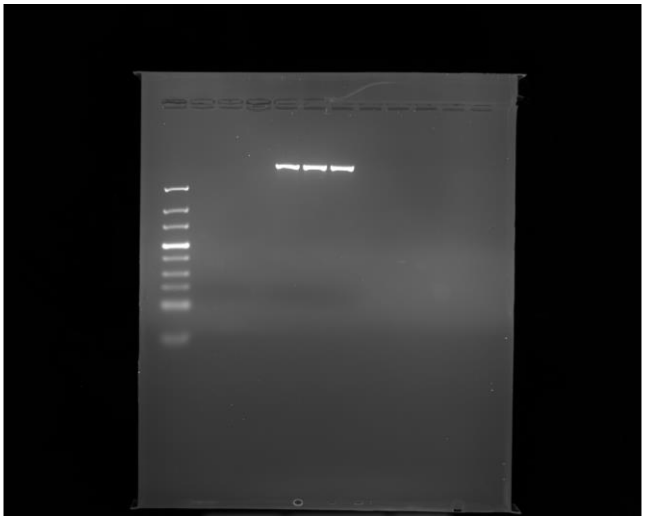

**Supplementary Figure S3**. HMGB1 signaling, as presented by IPA

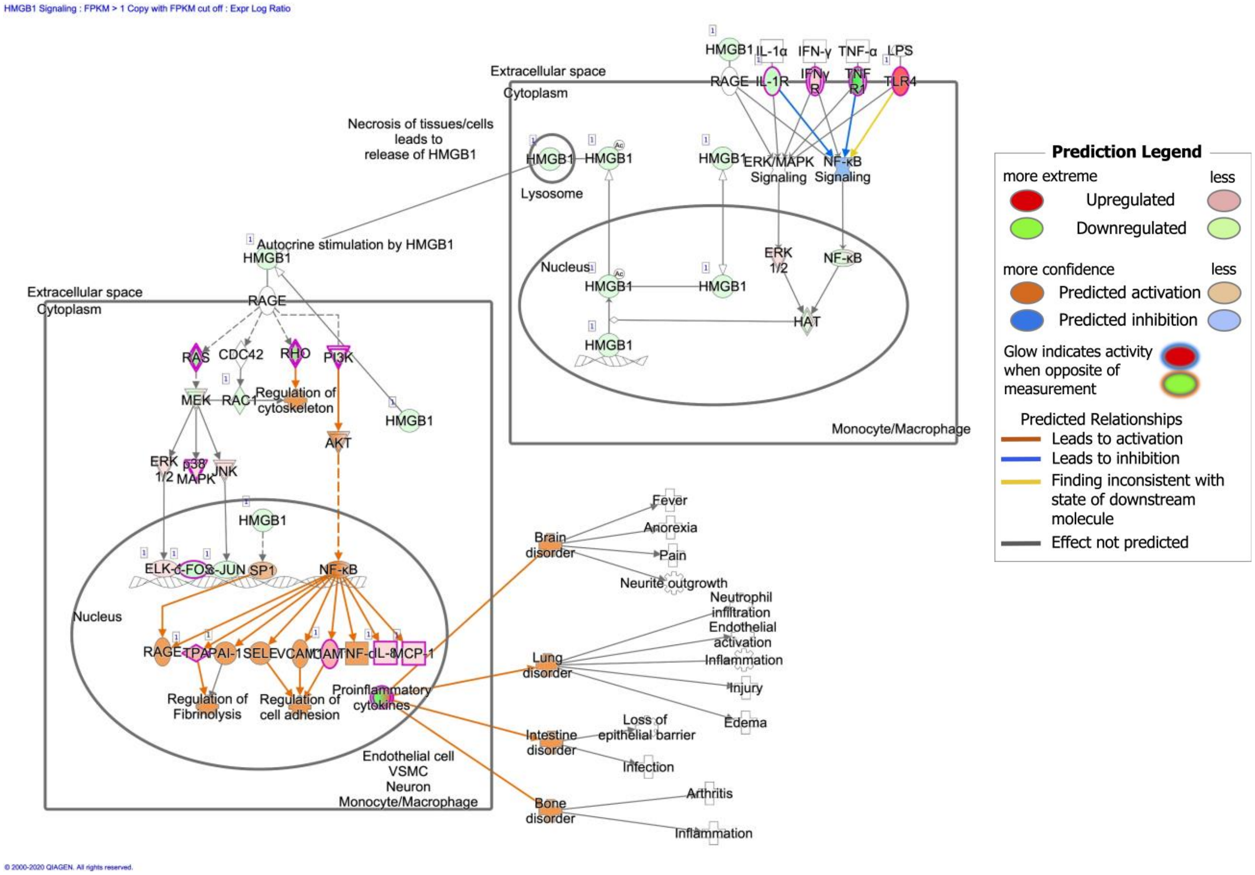

**Supplementary Figure S4**. Cell cycle Control of Chromosomal Replication, as presented by IPA

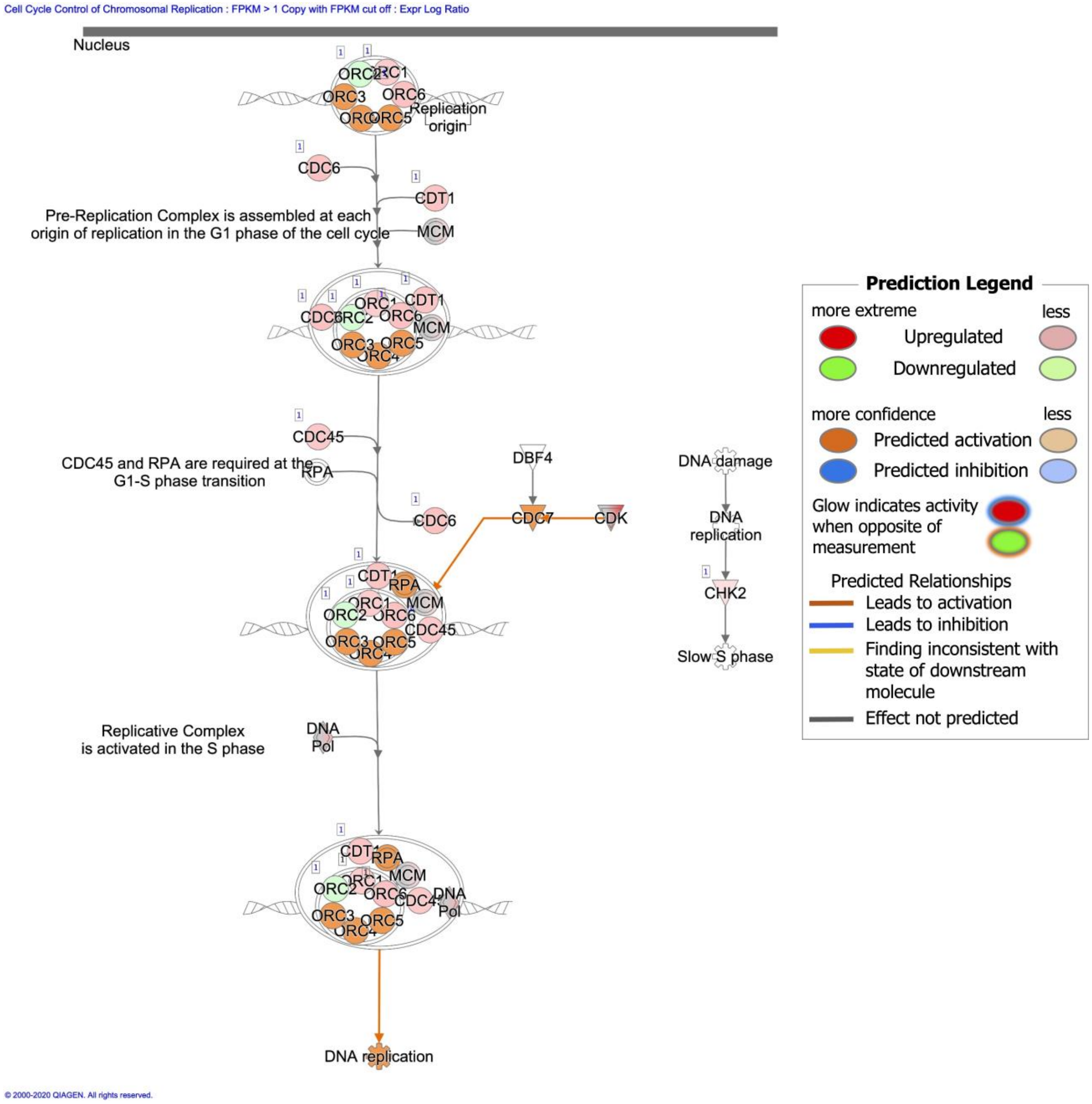

**Supplementary Figure S5**. Role of PKR in Interferon Induction and Antiviral Response, as presented by IPA

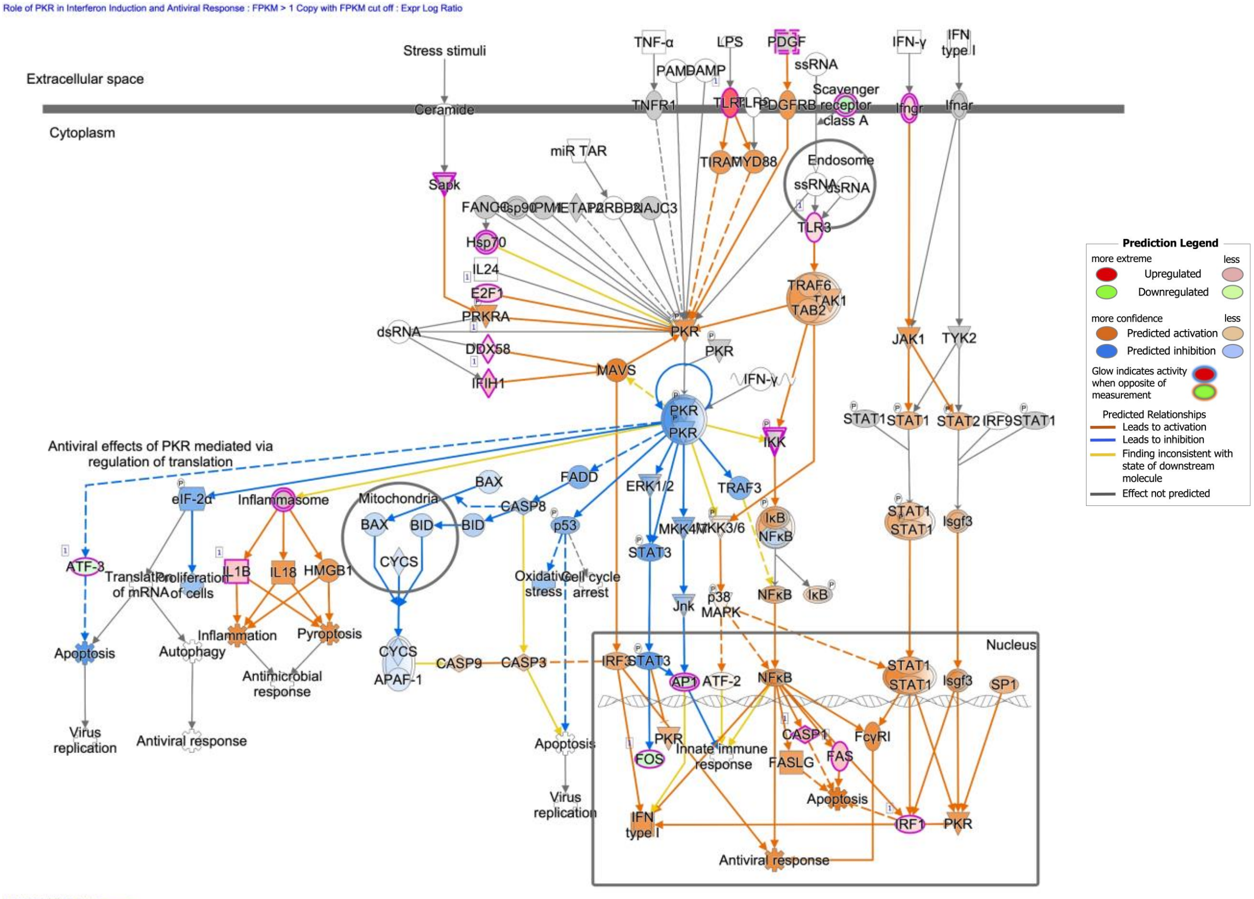


© 2000-2020 QIAGEN. All rights reserved.

**Supplementary Figure S6**. SPINK1 General Cancer Pathway, as presented by IPA

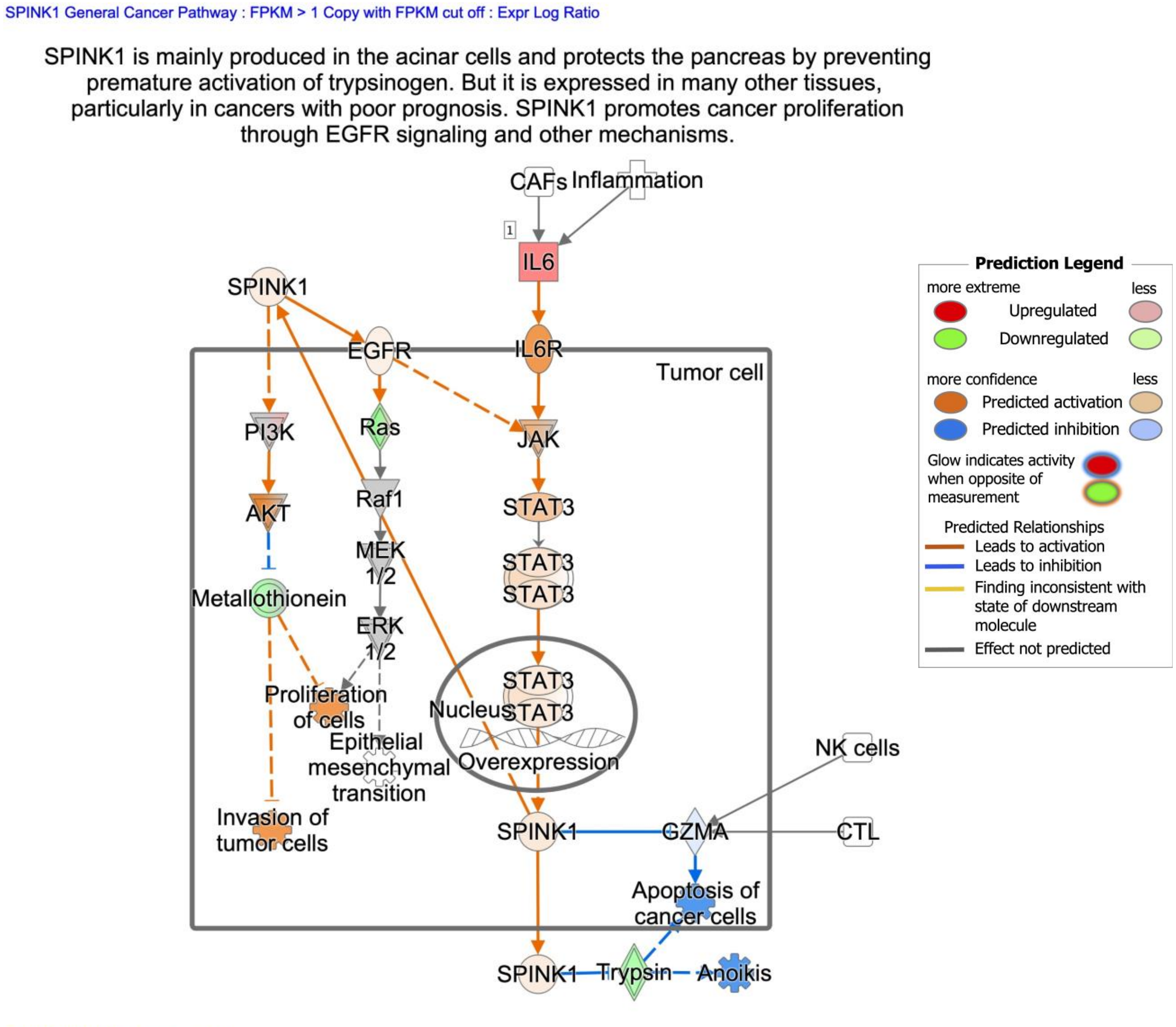


© 2000-2020 QIAGEN. All rights reserved.

**Supplementary Figure S7**. Tec Knase Signaling, as presented by IPA

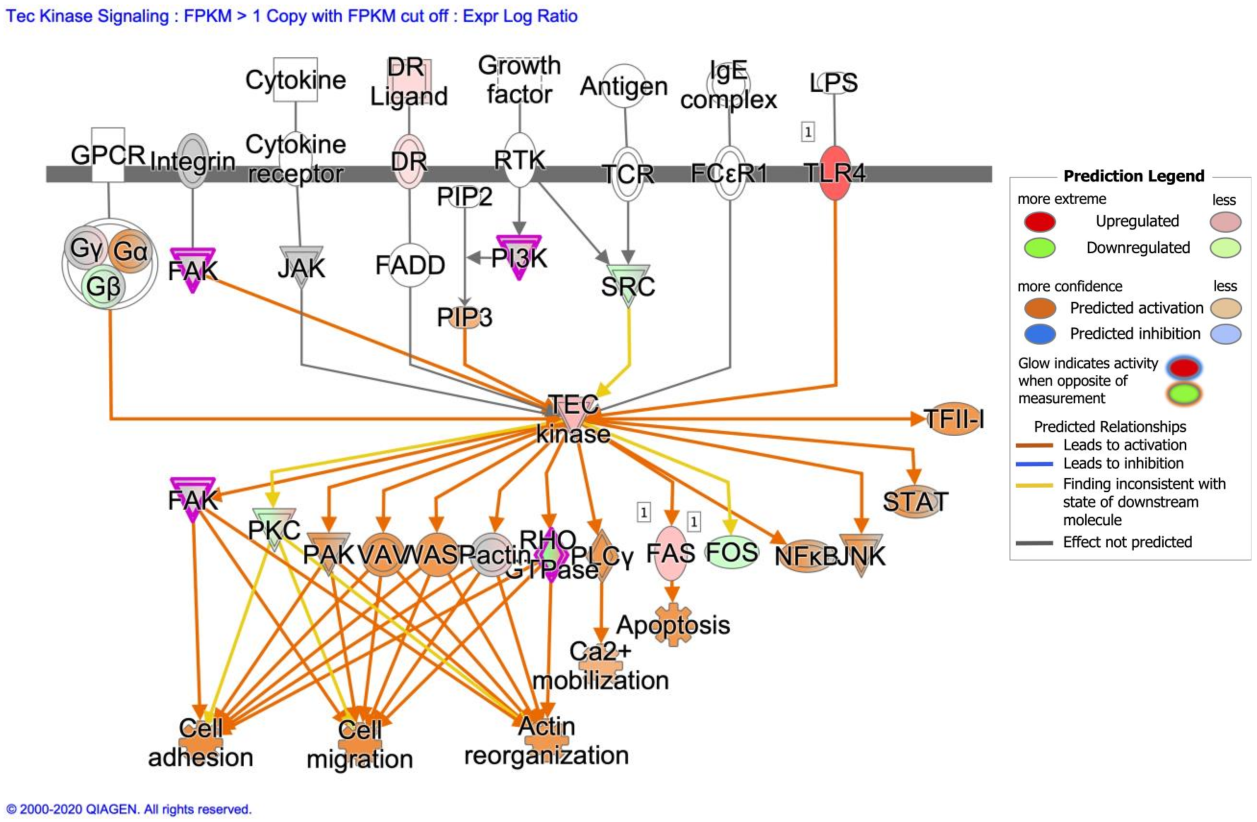

**Supplementary Figure S8**. Regulation of the Epithelial-Mesenchymal Transition by Growth Factors Pathway, as presented by IPA

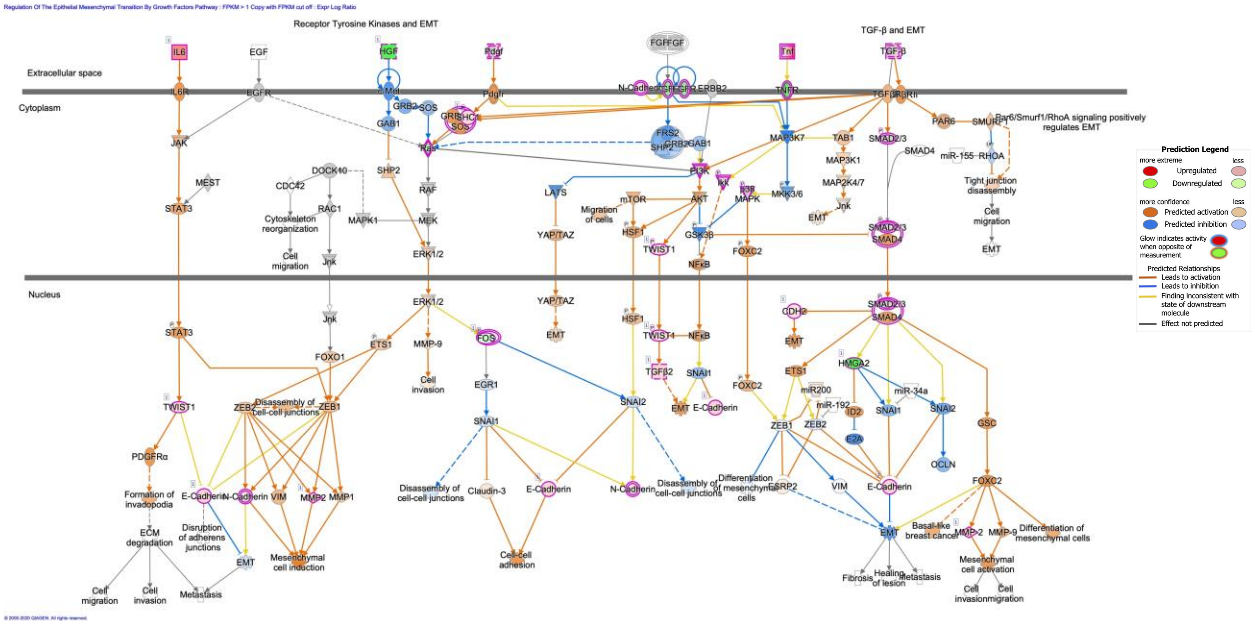

**Supplementary Figure S9**. Wnt/β-catenin Signaling, as presented by IPA

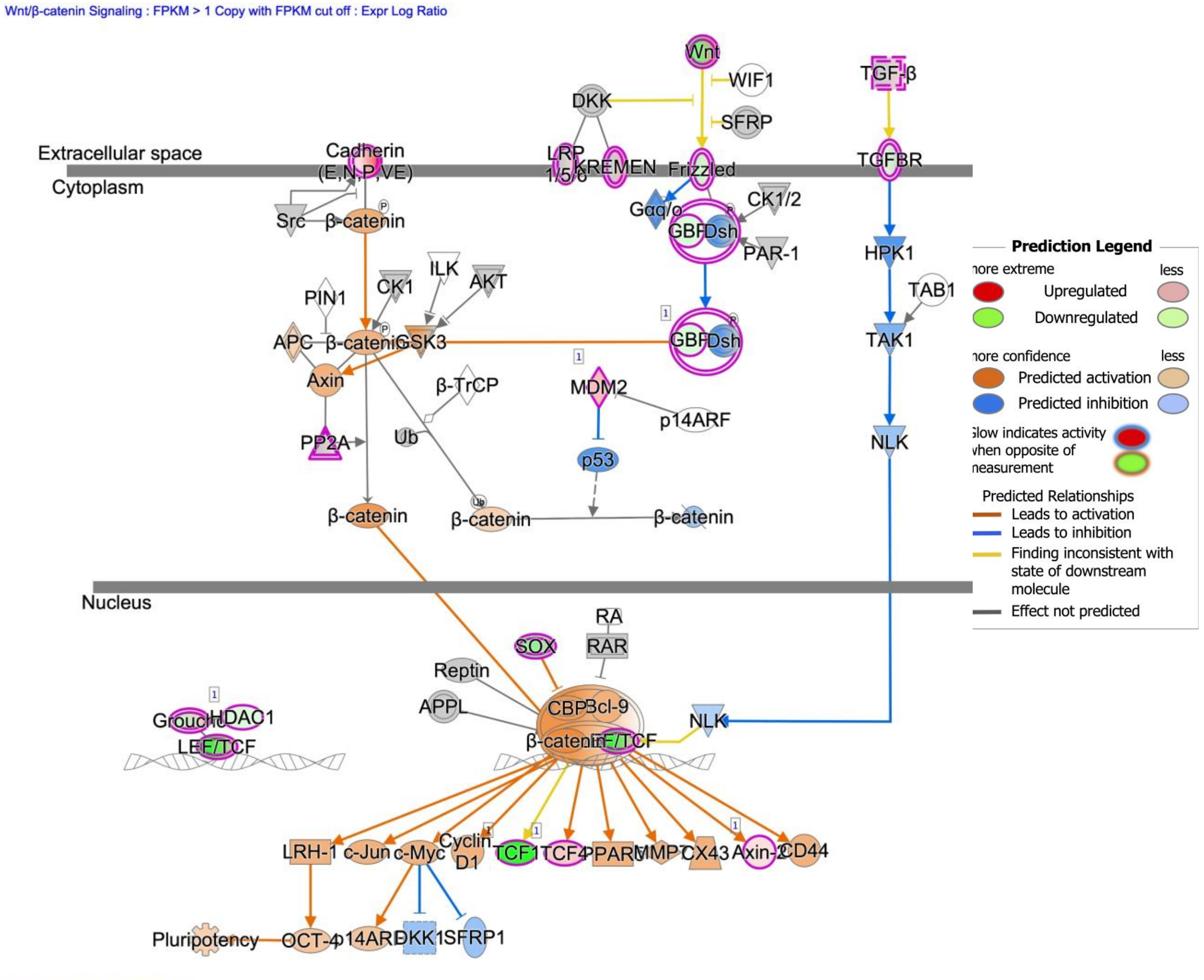


© 2000-2020 QIAGEN. All rights reserved.

**Supplementary Figure S10**. Sphingosine-1 phosphate Signaling, as presented by IPA

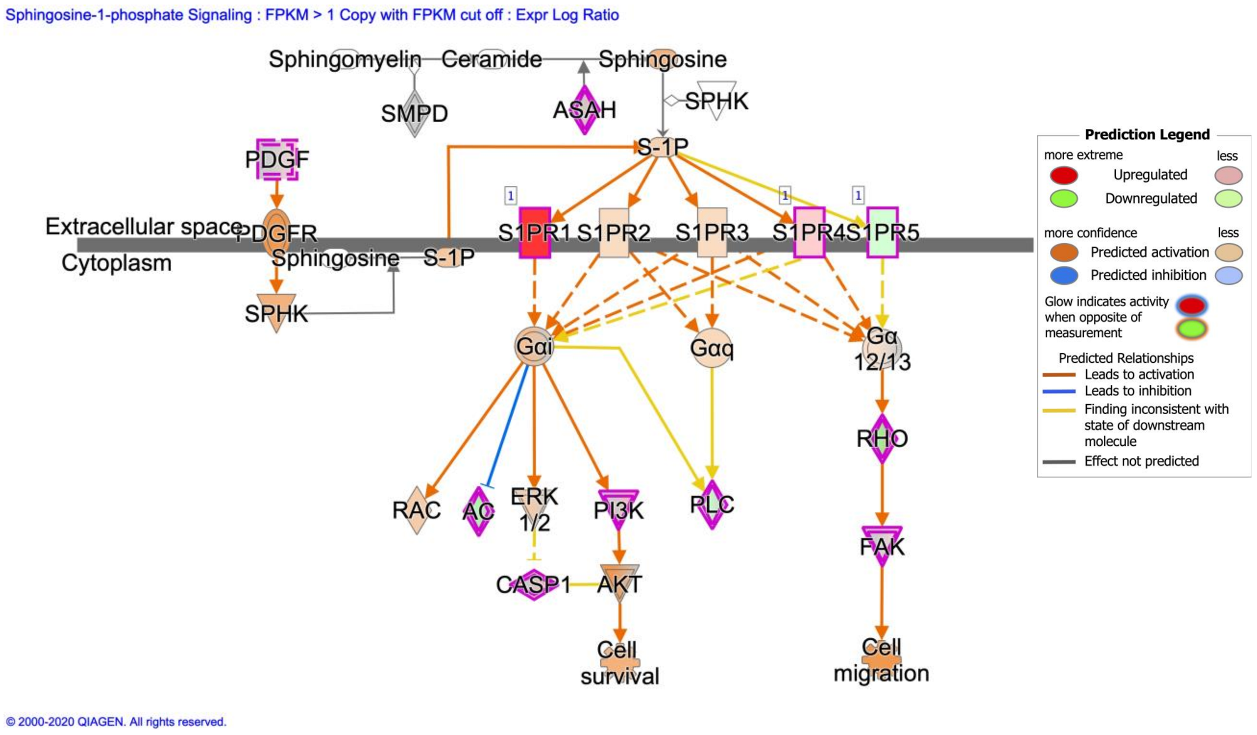

**Supplementary Figure S11**. Cholesterol Biosynthesis pathways, as presented by IPA

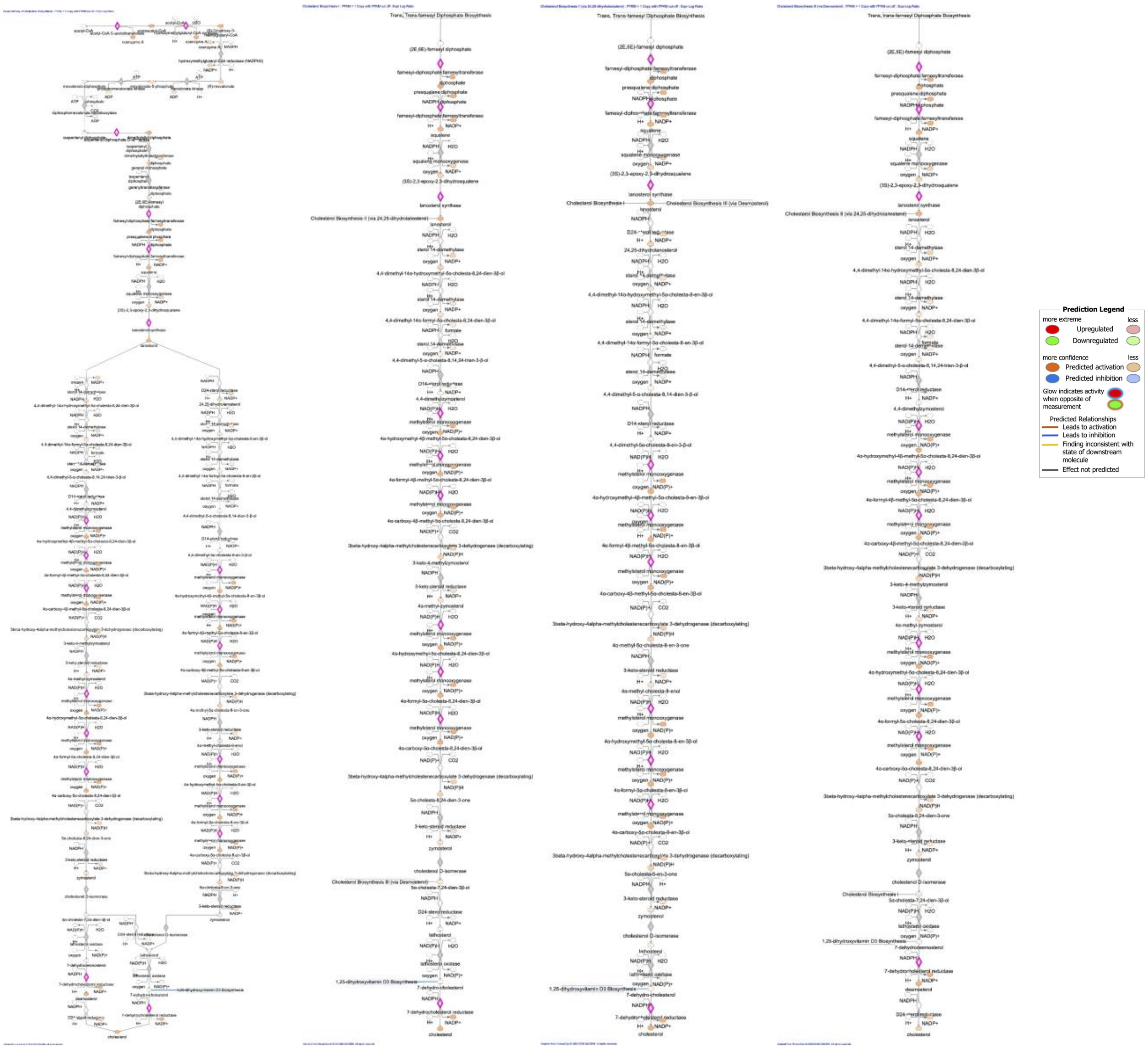

**Supplementary Figure S12**. TREM1 signaling, as presented by IPA

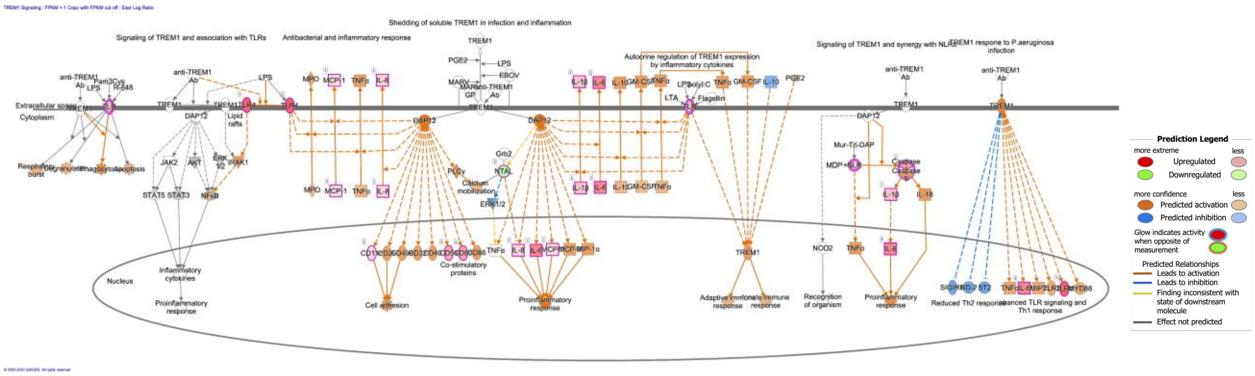

**Supplementary Figure S13**. IL-8 signaling, as presented by IPA

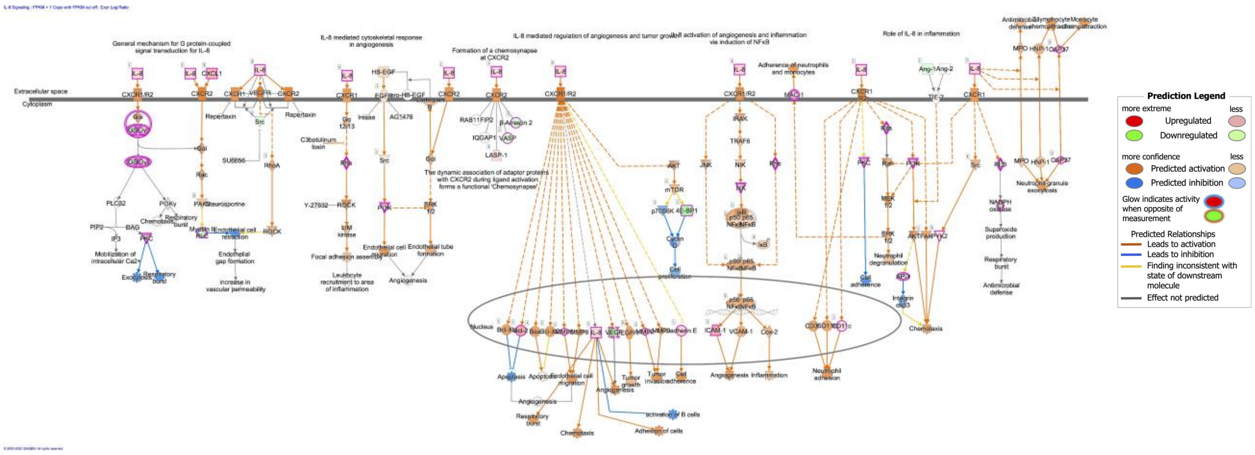

**Supplementary Figure S14**. Kinetochore Metaphase Signaling Pathway, as presented by IPA

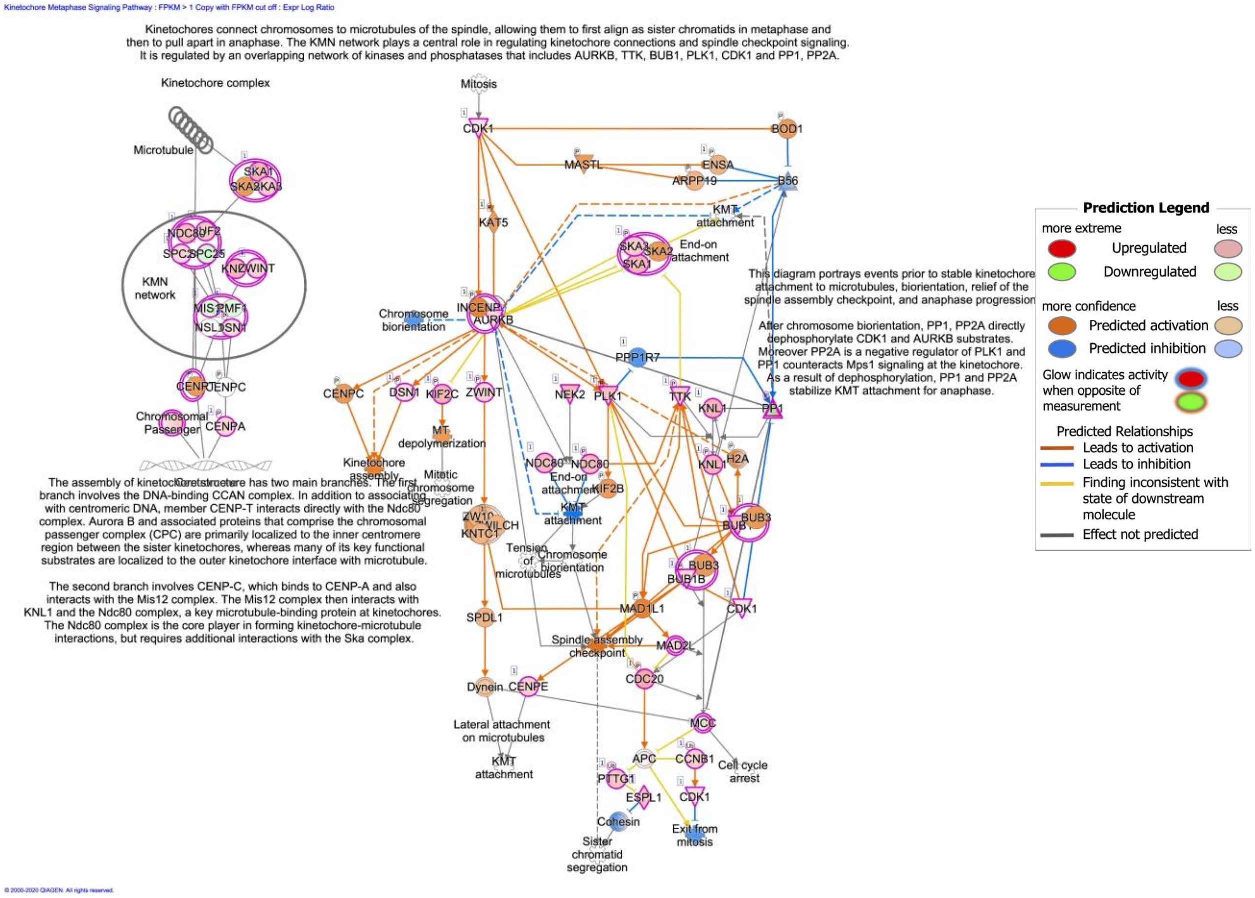

Supplement: Supplementary file 8 — Supplementary Information. [file 41598_2021_85930_MOESM8_ESM.pdf]
